# Long-range repulsion between chromosomes in mammalian oocyte spindles

**DOI:** 10.1126/sciadv.adq7540

**Published:** 2024-09-25

**Authors:** Colm P. Kelleher, Yash P. Rana, Daniel J. Needleman

**Affiliations:** ^1^ Department of Molecular and Cellular Biology, Harvard University, Cambridge, MA 02138, USA.; ^2^ John A. Paulson School of Engineering and Applied Sciences, Harvard University, Cambridge, MA 02138, USA.; ^3^ Center for Computational Biology, Flatiron Institute, New York, NY 10010, USA.

## Abstract

During eukaryotic cell division, a microtubule-based structure called the spindle exerts forces on chromosomes. The best-studied spindle forces, including those responsible for the separation of sister chromatids, are directed parallel to the spindle’s long axis. By contrast, little is known about forces perpendicular to the spindle axis, which determine the metaphase plate configuration and thus the location of chromosomes in the subsequent nucleus. Using live-cell microscopy, we find that metaphase chromosomes are spatially anti-correlated in mouse oocyte spindles, evidence of previously unknown long-range forces acting perpendicular to the spindle axis. We explain this observation by showing that the spindle’s microtubule network behaves as a nematic liquid crystal and that deformation of the nematic field around embedded chromosomes causes long-range repulsion between them.

## Teaser

Nematic elasticity plays a fundamental role in determining both spindle structure and chromosome positioning in oocyte spindles.

## INTRODUCTION

Chromosome segregation is a mechanical process, requiring precisely coordinated motion of micrometer-sized objects (chromosomes) through distances of tens of micrometers (the size of a typical metazoan cell) (*1*, *2*). The forces causing this motion are generated by the spindle, a structure comprising a network of microtubules—long, rigid polymers of the protein tubulin—in association with hundreds of additional proteins that modulate microtubule nucleation, polymerization/depolymerization, and interactions (*3*, *4*). Extensive previous work has identified specific molecules (e.g., molecular motors) and processes (e.g., microtubule depolymerization) that are required for some of the most important chromosome motions, including chromosome congression during prometaphase and sister chromatid separation in anaphase (*5*, *6*). Notably, all known force-generating processes in the spindle create forces parallel to microtubules, i.e., along the spindle axis. Forces perpendicular to the spindle axis are little studied, although they are key determinants of the metaphase plate configuration and thereby contribute to important cellular properties, including chromosomal nuclear localization and the probability of segregation errors (*7*, *8*).

In materials physics, continuum theories are commonly used to describe how forces and torques propagate through systems comprising large numbers of interacting molecules (*9*). For over a century, cell biologists have tried to apply these ideas to living structures, including the spindle (*10*–*12*). For much of this time, the experimental challenge of measuring material properties in vivo, in addition to the theoretical difficulty of applying continuum theories to far-from-equilibrium, living materials, prevented the development of quantitative models of spindle mechanics. Recent decades, however, have seen important advances: quantitative microscopy facilitates noninvasive characterization of the material properties of cellular structures (*13*–*15*), and progress in active matter physics allows more complete theoretical descriptions of far-from-equilibrium materials (*16*). By leveraging these developments, quantitative microscopy of spindles reconstituted in vitro from *Xenopus* egg extract revealed that the tendency of microtubules to locally align with one another (i.e., nematic elasticity) is crucial in determining the physical properties of these spindles (*13*, *17*). However, it remains unclear if a similar materials physics–based approach will succeed in describing the physical properties of spindles in intact living cells.

**Fig. 1.**
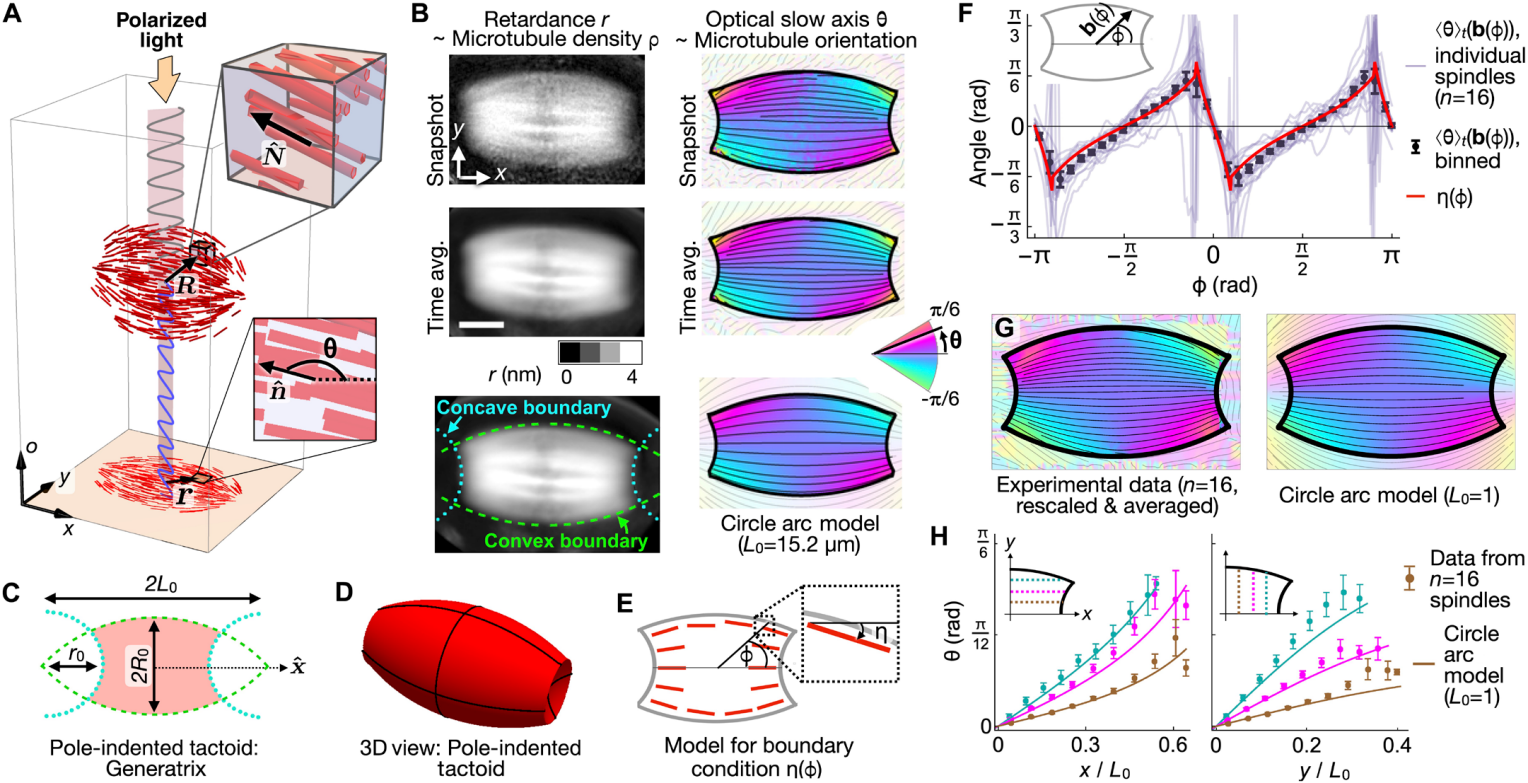
In MII mouse oocyte spindles, microtubule orientation in the interior is fully determined by surface geometry. (**A**) For a spindle with $\hat{x}⊥\hat{o}$, the retardance *r*(**r**) and slow axis angle θ(**r**) measured by LC-PolScope are related to the $\hat{o}$-projected density ρ(**R**) and director $\hat{N}\left(R\right)$. (**B**) Top and middle rows*:* Snapshots and time averages (500 frames, 40 min) of *r* and θ for an MII spindle with $\hat{x}⊥\hat{o}$. Scale bar, 5 μm. Bottom left: The spindle boundary is fitted to four circle arc segments that correspond to the convex and concave surfaces of the spindle (green dashed curves and cyan dotted curves, respectively). The green dashed circles intersect at (±*L*_0_,0), with *L*_0_ = 15.2 μm. Bottom right*:* The circle arc model with *L*_0_ = 15.2 μm closely matches the observed microtubule orientation in the spindle interior. (**C** and **D**) The geometric parameters *L*_0_, *R*_0_, and *r*_0_ specify a pole-indented tactoid. (**E**) On the convex [concave] regions of the spindle boundary, η(ϕ) is the angle tangent [perpendicular] to the best-fit pole-indented tactoid, where ϕ is the polar angle in the *xy* plane (inset). (**F**) The measured microtubule orientation at the spindle boundary 〈θ〉*_t_*[**b**(ϕ)] (points with error bars) closely follows the predicted form η(ϕ) (red solid curve). (**G**) Rescaling **r** → **r**/*L*_0_ allows averaging over spindles. (**H**) Horizontal and vertical line profiles of microtubule orientation for *n* = 16 spindles. For each spindle, data are rescaled and transformed into the first quadrant before binning. Predictions of the rescaled circle arc model (*L*_0_ = 1), which contains no fit parameters, are shown as bold curves. Error bars indicate SE.

**Fig. 2.**
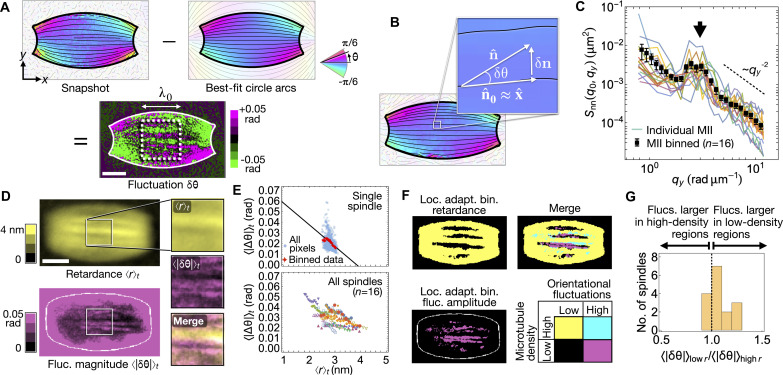
Orientational fluctuations in mouse oocyte spindles are highest in regions of low microtubule density. (**A**) Fluctuations are calculated by subtracting steady-state values of the microtubule orientation field from instantaneous values. Correlation functions are calculated in the white dashed box (side length λ_0_) where ${\hat{n}}_{0}\approx \hat{x}$. (**B**) In the dashed box, $\delta n\cdot {\hat{n}}_{0}\approx \delta n\cdot \hat{x}=0$ and, to lowest order in δθ, $\delta n=\delta \theta \;\hat{y}$. (**C**) Spatial correlation function *s*_nn_(*q*_0_, *q_y_*) for 16 spindles, where *q*_0_ = 2π/λ_0_ and *q_y_* is the wave vector component perpendicular to the spindle axis. For the smallest and largest *q_y_*, *s*_nn_(*q*_0_, *q_y_*) decays as $q_{y}^{-2}$; around $q_{y}^{*}=\left(3.0\pm 0.1\right)$ rad μm^−1^, a peak appears (black arrow). Error bars indicate SE. (**D**) Time-averaged retardance 〈*r*〉*_t_* (top, yellow) and orientational fluctuations 〈∣δθ∣〉*_t_* (bottom, purple). (**E**) Top: Near the center of the spindle, orientational fluctuations are negatively correlated with retardance. Black line shows linear fit, slope (−0.017 ± 0.007) rad nm ^−1^. Bottom*:* Orientational fluctuations are negatively correlated with retardance in all but one spindle. (**F**) Applying a local binarization filter to 〈*r*〉*_t_* and 〈∣δθ∣〉*_t_* reveals elongated regions of low microtubule density; orientational fluctuations are larger, on average, in low-density regions. (**G**) Histogram of the ratio of fluctuations in lower-density regions to fluctuations in higher-density regions; the average value of 〈∣δθ∣〉_low r_/〈∣δθ∣〉_high r_ over all spindles is 1.07 ± 0.02 (mean ± SE). Scale bars, 5 μm.

**Fig. 3.**
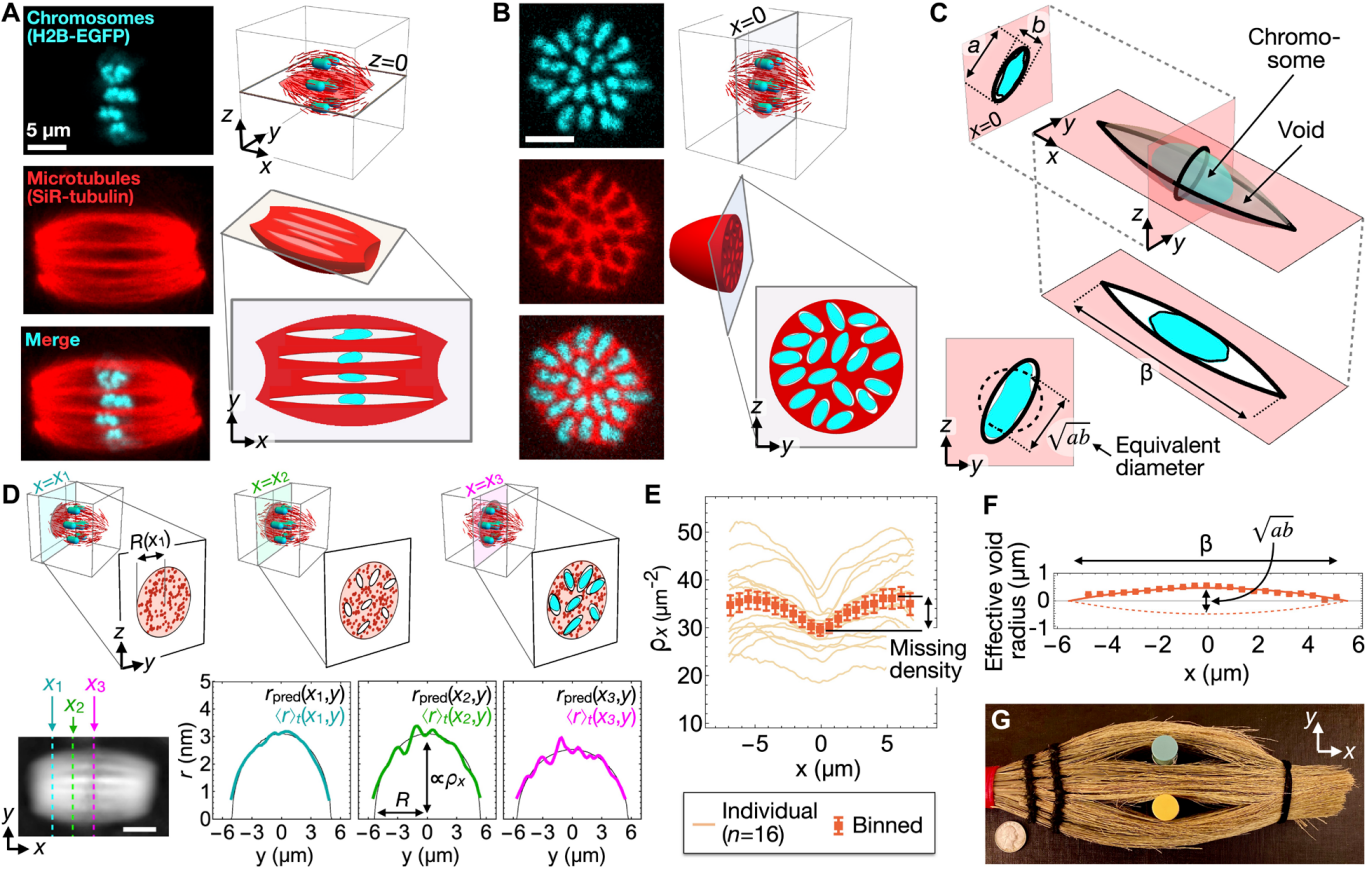
Chromosomes create tactoid-like voids in the microtubule network of MII spindles. (**A**) Confocal micrographs of the *z* = 0 plane. Microtubules are labeled with SiR-tubulin, chromosomes with H2B-EGFP. (**B**) In the metaphase plate (*x* = 0 plane), chromosome positions correspond to voids. (**C**) In the metaphase plate, the void cross-section approximates an ellipse with major and minor axes *a* and *b*. Inset: The tactoid of equal volume to the void shown has a circular cross section with diameter $\sqrt{\mathrm{ab}}$. (**D**) Top row: For spindles with $\hat{x}⊥\hat{o}$, the retardance profile *r*(*x_i_*, *y*) at a given position *x_i_* along the long axis can be fitted to *r*_pred_(*x_i_*, *y*) = 2*A*_0_ρ*_x_*(*x_i_*)[*R*(*x_i_*)^2^ − *y*^2^]^1/2^, where ρ*_x_*(*x_i_*) is the cross-sectional density and *R*(*x_i_*) is the spindle radius in the plane *x* = *x_i_* (text S3). Larger values of ρ*_x_*(*x_i_*) correspond to less area taken up by voids. Bottom row: Fits to 〈*r*〉*_t_*(*x_i_*, *y*) at positions *x*_1_, *x*_2_, and *x*_3_ along the spindle axis. (**E**) Microtubule cross-section density ρ*_x_* as a function of *x*. (**F**) The average void profile is well approximated by a circle arc (solid curve) with waist diameter $\sqrt{\mathrm{ab}}=\left(1.0\pm 0.1\right)\;\mu m$ and length β = (11 ± 1) μm (both 95% confidence interval). To aid visualization, the fit is also shown reflected in the $\hat{x}$ axis (dashed curve). (**G**) The formation of voids around compact inclusions is also seen when cylindrical lip gloss tubes are inserted into a straw broom head. U.S. quarter dollar coin included for scale. Error bars indicate SE; scale bars, 5 μm.

**Fig. 4.**
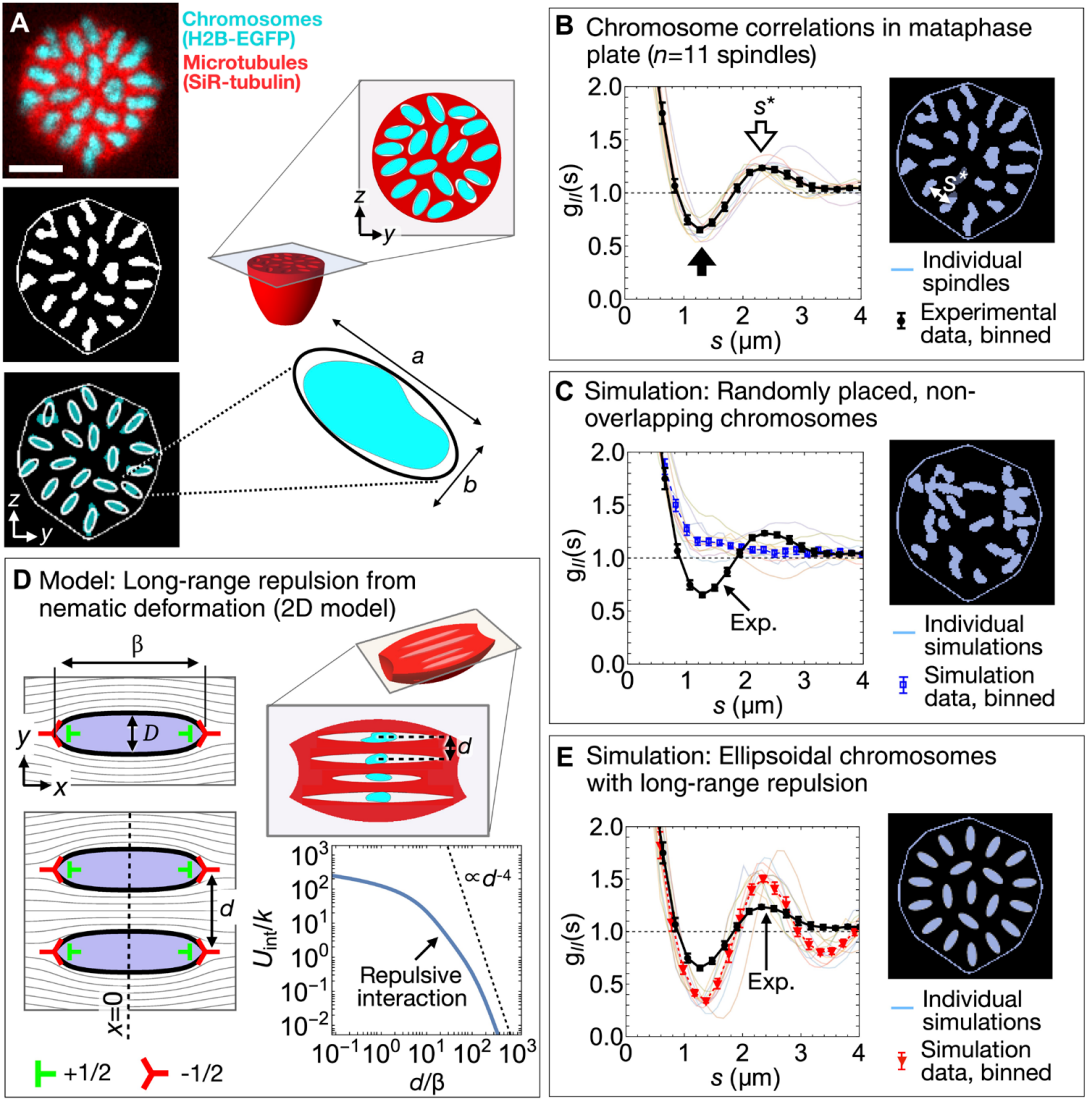
Metaphase plate configurations in MII spindles are consistent with chromosome repulsion over micrometer scales. (**A**) Top: Confocal micrograph of the metaphase plate, labeled as in Fig. 3. Middle: Binarization identifies *n*_chr_ = 20 chromosome sections. Spindle boundary in white. Bottom: Chromosome sections approximated by 20 identical ellipsoids, with *a* = 2.1 μm and *b* = 0.8 μm. Scale bar, 5 μm. (**B**) The average experimental correlation function *g_II_*(*s*) has a local minimum at (1.27 ± 0.04) μm (black arrow) and a local maximum at s^*^ = (2.4 ± 0.1) μm (white arrow) corresponding to the typical distance between chromosomes (inset image). Continuous colored curves show data *g_II_*(*s*) for *n* = 11 individual spindles; black points indicate the average. (**C**) Correlation functions *g_II_*(*s*) for simulated data of randomly placed 2D chromosome sections decay monotonically to 1. Each colored curve is obtained from a simulation initialized with one experimental dataset. Inset image: Snapshot of a simulation initialized with the spindle boundary and chromosome sections shown in (A). (**D**) Model for long-range repulsion between negative tactoid–like inclusions in a 2D nematic (text S10). Topological defect spacings within a row are determined by the geometric parameters β and *D*. The interaction potential *U*_int_/*k* is repulsive at all center-to-center distances *d*. In the limit *d*/β ≫ 1, *U*_int_ ∝ *d*^−4^ (dashed line). (**E**) Correlation functions *g_II_*(*s*) for simulated data of 20 ellipsoids interacting via a repulsive *U*(*s*) ∝ *s*^−5^ potential display a local minimum [maximum] at (1.32 ± 0.04) μm [(2.4 ± 0.1) μm]. Inset image: Simulation initialized with spindle boundary and chromosome shape parameters from (A). Error bars indicate SE.

To answer this question, we used live-cell microscopy to image metaphase II (MII) spindles in mammalian oocytes. Our results indicate that, like in vitro-reconstituted spindles, nematic elasticity governs properties such as the spindle’s barrel-like shape and the organelle-scale pattern of microtubule orientation in the spindle interior (Fig. 1). Unlike in vitro-reconstituted spindles, however, oocyte spindles contain higher-order structures that we call voids. These voids are elongated holes in the microtubule network, each centered on a single condensed chromosome, and they disrupt the uniform propagation of torque in the spindle bulk (Figs. 2 and 3). Further investigation of the void configurations revealed a surprising discovery: Voids and their associated chromosomes are locally ordered (i.e., spatially anti-correlated) within the metaphase plate. This observation implies the existence of a previously unknown force that acts to separate chromosomes from one another in the direction perpendicular to the spindle axis (Fig. 4). Our models and simulations suggest that this force is due to deformation of the nematic field around the voids/chromosomes, which leads to repulsion between them.

From a theoretical standpoint, our work suggests that nematic elasticity plays a fundamental role in determining both spindle structure and chromosome organization in large spindles across organisms and cell types. From an applied perspective, the image analysis pipeline we have developed provides a noninvasive, quantitative assessment of the microtubule network in living spindles, and our techniques could potentially be leveraged in in vitro fertilization clinics to select oocytes with the greatest developmental potential.

## RESULTS

### Steady-state microtubule orientation in the spindle interior is controlled by nematic elasticity

Due to their large sizes and long steady-state lifetimes, MII spindles in mouse oocytes are an ideal model system in which to study spindle self-organization [text S1; (*18*–*20*)]. To characterize the structure and dynamics of the microtubule network in mouse oocyte spindles, we acquired LC-PolScope movies (Fig. 1A; Materials and Methods). LC-PolScope is a label-free quantitative polarization microscope that simultaneously measures the optical retardance *r*(**r**, *t*) and optical slow axis θ(**r**, *t*) at a given time *t* and each position **r** in a two-dimensional (2D) image (*18*). These measurements provide quantitative information regarding the coarse-grained microtubule cross-sectional density ρ(**R**, *t*) and the nematic director $\hat{N}\left(R,t\right)$, both defined at every point **R** in 3D space as well as in time (*21*): If the spindle long axis $\hat{x}$ is perpendicular to the optical axis $\hat{o}$

$$r\left(r,t\right)\approx A_{0}\int_{T}‍\rho \left(R,t\right)\;do;\hat{n}\left(r,t\right)\equiv \left[\text{cosθ}\left(r,t\right),\text{sinθ}\left(r,t\right)\right]\approx \frac{\int_{T}‍\hat{N}\left(R,t\right)do}{\left\|\int_{T}‍\hat{N}\left(R,t\right)do\right\|}$$ (1)

where the integrals are taken over the optical axis $\hat{o}$, the constant *A*_0_ ≈ 7.5 nm ^2^ characterizes the retardance contribution of a single microtubule, *T* is the sample thickness along the optical axis, and the 2D vector $\hat{n}\left(r,t\right)$ is the normalized projection of $\hat{N}\left(R,t\right)$ into the LC-PolScope image plane [Fig. 1A; texts S2 and S3; (*22*–*24*)].

We first used LC-PolScope movies to determine the relationship between the surface geometry of MII oocyte spindles and microtubule orientation in the spindle interior. To characterize surface geometry, we identified spindle boundaries from time-averaged retardance images, 〈*r*〉*_t_*, calculated in a spindle-referenced coordinate system where $\hat{z}=\hat{o}$, $\hat{y}=\hat{z}\times \hat{x}$, and the origin is fixed at the center of the spindle (Fig. 1B, left column, and Materials and Methods). We find that those portions of the spindle boundary that are furthest from the central axis are well fitted by a pair of circle arcs that intersect at “virtual poles” at (±*L*_0_,0), outside the spindle boundary (Fig. 1B, bottom left, dashed green curves). We fitted the remaining portions of the boundary, near the spindle poles, to circle arcs with radius *r*_0_ and centered at (±*L*_0_,0) (Fig. 1B, bottom left, dotted cyan curves). We found that this family of shapes provides better fits to the empirically measured spindle boundaries than other trial fitting forms, for instance, ellipses or rectangles (Fig. S4). Moreover, the spindle shape parameters obtained by fitting LC-PolScope images are similar (within ∼10%) to those found when the same procedure is applied to confocal slices containing the spindle long axis (Fig. S5). For each spindle we imaged, the closed curve defined by the best-fit circle arcs (Fig. 1C) may be rotated around $\hat{x}$ to generate a form we call a “pole-indented tactoid” that approximates the 3D shape of the spindle (Fig. 1D). This morphology is highly reminiscent of the tactoid and tactoid-like shapes seen in both experiments and simulations of nematic liquid crystal droplets (*25*–*27*).

We next examined how microtubule orientation at the spindle boundary depends on surface geometry. We found close agreement between the observed microtubule orientation and a purely geometrical model in which microtubules lie tangent to the spindle’s convex surface and perpendicular to its concave (polar cap) surfaces (Fig. 1, E and F). Thus, at the spindle surface, microtubules appear to obey a “strong anchoring” boundary condition (*28*). In tactoid-shaped nematic droplets with strong anchoring, the director is predicted to lie tangent to the unique family of circle arcs that intersect at (±*L*_0_,0,0) (*29*, *30*). To test whether this is the case in MII spindles, we compared the time-averaged slow axis images 〈θ〉*_t_* to the predictions of the circle arc model (Materials and Methods). For individual spindles, we find good agreement between the observed pattern of microtubule orientation and that predicted by the model (Fig. 1B, bottom right; text S5). To further probe the validity of the circle arc model, we investigated whether steady-state orientation fields from different spindles collapse onto a master field when they are properly rescaled, as that model predicts they should. Using previously measured values of *L*_0_ (and no additional fit parameters), we rescaled the orientation field of all spindles, 〈θ〉*_t_*(**r**) → 〈θ〉*_t_*(**r**/*L*_0_), and observed excellent data collapse (Fig. 1, G and H). Together, these results provide strong evidence for a model of spindle self-organization in which microtubule orientation is determined by a well-defined anchoring condition on the spindle boundary, together with a tendency for microtubules in the spindle interior to locally align with each other, i.e., nematic elasticity.

### Nematic torque propagation is disrupted by regions of low microtubule density

Since the steady-state orientation of microtubules in MII spindles is well described by nematic liquid crystal physics, we next investigated if such a model can also describe the fluctuations in microtubule orientation around that steady state, a phenomenon that has long been used to probe how nematic materials transmit torque (*28*, *31*). More recently, fluctuation analysis was used to show that the microtubule network of in vitro reconstituted *Xenopus* egg extract spindles behaves as an active nematic material (*13*). To determine if a similar approach can be applied to spindles in living oocytes, we first subtracted the best-fit 2D director field, ${\hat{n}}_{0}\left(r\right)$, from the instantaneous field $\hat{n}\left(r,t\right)$ to calculate the fluctuations $\delta n\left(r,t\right)=\hat{n}\left(r,t\right)-{\hat{n}}_{0}\left(r\right)$ in a box of side length λ_0_ = 8 μm placed at the center of the spindle (Fig. 2A). Fluctuations take a particularly simple form in this region since, to lowest order in δθ, $\delta n\approx \delta n_{y}\hat{y}$ and δ*n_y_* = δ(sinθ) ≈ δθ (Fig. 2B). To quantify the fluctuation pattern, we plotted the equal-time correlation function, *s*_nn_(*q*_0_, *q_y_*), as a function of the wave vector component *q_y_* perpendicular to the spindle axis, with the parallel component fixed at the lowest available mode *q*_0_ = 2π/λ_0_ (Materials and Methods). For the lowest and highest values of *q_y_*, *s*_nn_(*q*_0_, *q_y_*) displays behavior consistent with the inverse square power law predicted by active nematic theory and observed in previous experiments on reconstituted *Xenopus* spindles (Fig. 2C and text S6). At intermediate value of *q_y_*, however, *s*_nn_(*q*_0_, *q_y_*) displays a prominent feature neither predicted by theory nor observed in *Xenopus* extract spindles: a peak centered at $q_{y}^{*}$ = (3.0 ± 0.1) rad μm^−1^, corresponding to a real-space wavelength λ* = 2π/$q_{y}^{*}$ = (2.1 ± 0.1) μm.

To elucidate the origins of the anomalous behavior of *s*_nn_(*q*_0_, *q_y_*), we next explored the relationship between orientational fluctuations and microtubule density. We observed that the time-averaged orientational fluctuation magnitude 〈∣δθ∣〉*_t_* was negatively correlated with the time-averaged retardance 〈*r*〉*_t_* in 15 of 16 spindles (Fig. 2, D and E, and text S7). To investigate the basis of this negative correlation, we used a local binarization filter (Materials and Methods), which revealed that spindles contain elongated regions with low microtubule density (Fig. 2F) in which orientational fluctuations are larger (Fig. 2G). In these regions, nematic elasticity–dominated torque transmission is apparently disrupted, leading to the characteristic peak in *s*_nn_(*q*_0_, *q_y_*) (Fig. 2C and texts S6.5 and 6.6).

### Chromosomes cause tactoid-like voids in the microtubule network

To understand in greater detail the origins of these micrometer-scale density inhomogeneities and how they might affect orientational fluctuations, we labeled chromosomes by expressing H2B-EGFP and microtubules by SiR-tubulin staining, and used 3D confocal microscopy to image the internal structure of living oocyte spindles (Materials and Methods). Confocal micrographs show that the microtubule network is a contiguous material perforated by voids (i.e., regions of low microtubule density) surrounding each embedded chromosome (Fig. 3, A and B). Consistent with recent results demonstrating that condensed chromosomes are microtubule impermeable (*32*), void cross-sections in the metaphase plate (i.e., the *x* = 0 plane) closely follow chromosome boundaries and may be approximated as ellipses with long and short axes *a* and *b*, respectively, where *a*/*b* ≈ 2.5 (text S8). In the $\hat{x}$ direction, the voids extend much further than chromosomes, along most of the length of the spindle (Fig. 3C).

We were not able to use the micrographs to quantify individual void profiles along $\hat{x}$ because, for much of their lengths, void widths are smaller than the microscope’s resolution. Instead, we used LC-PolScope data to infer the average void profile along $\hat{x}$. To do this, we fit the microtubule cross-sectional density ρ*_x_*(*x*) = 〈ρ(**R**, *t*)〉_*y*,*z*,*t*_ as a function of position *x* along the spindle long axis (Fig. 3, D and E, and text S3). The density profile ρ*_x_*(*x*) reaches a minimum at the metaphase plate and a maximum near the poles (Fig. 3E). By assuming that all of this “missing” density in the central spindle is due to voids, we infer that ∼10% of the total volume, and ∼15% of the metaphase plate area, of MII spindles is taken up by voids. Assuming further that all spindles contain *n*_chr_ = 20 voids (one per chromosome), we find that average void profiles along $\hat{x}$ are well approximated by circle arcs with waist diameter around 1 μm (Fig. 3F), which may be interpreted as the geometric mean of the long and short axes of the void’s *x* = 0 cross-section, $\sqrt{\mathrm{ab}}$ (Fig. 3C, bottom left inset). The simplest 3D shape that would generate such a missing retardance profile is the tactoid generated by revolving a circle arc of chord length β about its chord, which would have a circular cross-section in the metaphase plate (*a* = *b*). Since the voids in MII spindles have noncircular *x* = 0 cross-sections (*a* ≠ *b*), their 3D shapes are not true tactoids, but rather are “tactoid-like” in the sense that their average profile along $\hat{x}$ is a circle arc.

Tactoid-shaped holes have been observed previously in both synthetic and biological nematics, and are known as “negative tactoids” or “atactoids” (*33*–*35*). To minimize deformation energy, these structures tend to spontaneously align with the director (*36*–*38*). A macroscopic example of this phenomenon can be observed in a straw broom head whose loose end is tied with an elastic band. This system constitutes a confined, quasi-2D, nematically ordered material with linear dimensions ∼10 cm and director $\approx \hat{x}$ (Fig. 3G). When plastic lip gloss tubes (∼1 cm in diameter) are inserted near the broom head mid-plane, negative tactoid–like voids spontaneously form. As in oocyte spindles, in the $\hat{y}$ direction, the void diameter equals the inclusion size; in the $\hat{x}$ direction, voids extend across lengths comparable to the system size.

### Chromosomes/voids are locally ordered in the metaphase plate

To investigate the chromosome/void configuration in the direction perpendicular to the spindle axis, we binarized images of the metaphase plates of several spindles (Fig. 4A). To detect correlations between chromosome positions, we use the pair correlation function *g_II_*(*s*), which quantifies the average correlation between the pixel value *I* at pairs of points separated by a distance *s* in the metaphase plate (text S8). At separations much less than the smaller chromosome dimension (*s* ≪ *b* ≈ 1 μm), *g_II_*(*s*) ≫ 1; this reflects the fact that a white pixel is very likely to immediately neighbor other white pixels. At larger separations, we observe a local minimum at (1.27 ± 0.04) μm, indicating the presence of a ring around each chromosome that is depleted of other chromosomes, and a local maximum at s* = (2.4 ± 0.1) μm corresponding to a ring enriched in chromosomes (Fig. 4B, black and white arrows; uncertainty given by empirical bootstrapping). To test whether these features of *g_II_*(*s*) can be explained by steric repulsion (that is, the tendency of chromosomes to avoid spatial overlap), we ran a Monte Carlo simulation that takes as inputs the experimentally determined set of binarized chromosome sections and re-arranges them into a random configuration while avoiding overlap (text S9). These configurations appear strikingly different to the experimentally observed ones, and the corresponding *g_II_*(*s*) lacks local maxima and minima (Fig. 4C). These simulations imply that steric repulsion is insufficient to produce the locally ordered chromosome configurations that we observe.

Local ordering of chromosomes and their associated voids also explains the previously noted peak in *s*_nn_(*q*_0_, *q_y_*) (Fig. 2C), since large fluctuations concentrated in regularly spaced voids cause a peak in the orientational correlation function (text S6). Consistent with this interpretation, the characteristic spacing between voids, s* = (2.4 ± 0.1) μm, is similar to the position of the correlation function peak, λ* = (2.1 ± 0.1) μm.

### Observed chromosome configurations are consistent with long-range repulsion from deformation of the nematic field

We next turned to the origin of the forces responsible for chromosome ordering in the metaphase plate. In liquid crystal physics, it is well established that deformation of the nematic field around micrometer-size inclusions can create long-range forces that cause the inclusions to self-organize into structured arrays (*39*, *40*). We therefore hypothesized that, in MII spindles, a similar force might cause the regularly spaced chromosome configurations we observe. To explore this effect, we constructed an analytically tractable 2D model in which the void surrounding a chromosome is represented as a topological quadrupole (with zero net charge) made up of two +1/2 and two −1/2 defects (Fig. 4D, top left). In this model, void boundaries are defined as those integral curves of the director that pass through the outer pair of −1/2 defects. We used a defect quadrupole because it is the simplest defect configuration that qualitatively reproduces the deformation field we expect voids to generate in the surrounding nematic. In our construction, the length β and width *D* of the void uniquely determine the spacing of the defects within a row and thus, for an isolated void, the orientation field everywhere in space (text S10). For a pair of parallel, director-aligned voids whose centers lie along a line perpendicular to the far-field director $\hat{x}$, the deformation-induced interaction potential *U*_int_(*d*) decays monotonically for all values of β, *D*, and center-to-center separation *d*, implying the existence of a repulsive force between 2D voids, independent of the details of void geometry (Fig. 4D). In the far-field limit *d*/β ≫ 1, the interaction potential is proportional to *d*^−4^, identical to the form of far-field interaction between aligned electric quadrupoles in one of the best-known scalar field theories, 2D electrostatics [text S10; (*41*)].

While we were able to construct an analytical solution for the interaction between aligned voids in 2D, we lack an equivalent theory for the 3D voids we observe in oocyte spindles. By analogy with 3D electrostatics, we might expect the far-field potential to be proportional to *s*^−5^ (*42*), but since 3D nematic elasticity is not a scalar field theory, we do not know of any formal argument that guarantees that this form of interaction will be preserved. In any case, the far-field limit does not apply to the observed void geometry, since *s*/β < 1. We therefore performed a series of simulations where chromosome/void sections interact via four different long-ranged repulsive potentials: the 2D interaction potential [*U*_int_(*s*)], the far-field 3D electrostatic quadrupole interaction potential (∼*s*^−5^), as well as two other commonly used long-ranged repulsive potentials, inverse cube (∼*s*^−3^) and Yukawa (∼*s*^−1^*e*^−*s*/λ*_B_*^, where λ*_B_* is a parameter that sets the length scale of the repulsion) (text S9). In each simulation, chromosome sections are represented by ellipses, and the ellipse geometry and metaphase plate boundary are determined from a specific experimental dataset. We find that the specific form of the interaction does not qualitatively affect chromosome ordering: All simulations produce configurations similar to the experimentally observed one, with features such as an outer ring of ∼15 mostly radially oriented chromosomes/ellipses, and local extrema of *g_II_*(*s*) near 1.3 and 2.4 μm (Fig. 4E and text S9). Thus, our observations of local ordering of chromosomes in the metaphase plate are consistent with the presence of long-range repulsion arising from deformation of the 3D nematic field of microtubule orientation.

## DISCUSSION

Here, we have demonstrated that chromosomes are locally ordered in the metaphase plate of MII mouse oocytes, implying the existence of repulsive interchromsomal forces acting perpendicular to the spindle axis (Fig. 4). The micrometer-scale distances between chromosome surfaces (text S9) are far larger than can be accounted for by known forces, such as those arising from electrostatic repulsion (*43*) or steric interactions between chromosome-associated proteins (*44*). To explain this observation, we proposed a mechanism whereby distortion of the microtubule network around chromosomes causes repulsion between them. Our model relies on the key assumption that stress and torque propagate through the microtubule network according to the predictions of nematic elasticity, i.e., that the microtubule network has the mechanical properties of a nematic liquid crystal. This assumption is consistent with several other observations of mouse oocyte spindles, in particular the shape of the spindle boundary (Fig. 1, B to F), the steady-state pattern of microtubule orientation in the spindle interior (Fig. 1, G and H), the functional form of spatial correlations of orientational fluctuations (Fig. 2C), and the appearance of director-aligned, tactoid-like voids around embedded chromosomes (Fig. 3). Consistent with our findings, previous work in *Xenopus* egg extract (*13*, *17*) and human tissue culture cells (*21*) showed that nematic models accurately predict several aspects of spindle structure and dynamics in those systems also. Together, these results suggest that nematic elasticity plays a fundamental role in determining both spindle structure and chromosome organization in large spindles across organisms and cell types.

## MATERIALS AND METHODS

### Oocyte collection and in vitro maturation

All mouse care and use was approved by the Institutional Animal Care and Use Committee at Harvard University (approval #11-16). We obtained oocytes from B6C3F1/J mice (Jackson Labs #100010) by adapting previously described protocols (*45*). We injected female mice, aged 8 to 12 weeks, with 7.5 international units pregnant mare serum gonadotrophin (ProspecBio) and harvested germinal vesicle (GV)–stage oocytes 44 to 48 hours later; euthanasia of mice was via carbon dioxide asphyxiation followed by cervical dislocation. We performed ovary dissection and oocyte collection in commercially available Advanced KSOM culture medium (EmbryoMax) to which we add bovine serum albumin (BSA; 4 g/liter; Sigma) and a meiotic inhibitor cocktail that has been shown to minimize damage to in vitro matured oocytes (*46*): 10 μM 3-isobutyl-1-methylxanthine (Sigma) and 50 μM *N* ^6^,2′-O-dibutyryladenosine 3′,5′-cyclic monophosphate sodium salt (Sigma). We removed most cumulus cells by gentle pipetting through a 100-μm Stripper tip (Origio) that we attached to a mouth pipette using ultraviolet (UV)–cure glue (Norland). We rinsed the oocytes through several droplets of inhibitor-free medium [Advanced KSOM + BSA (4 g/liter)] and matured them in a 200-μl droplet of inhibitor-free medium covered with several mm of LiteOil (EmbryoMax) to prevent evaporation. Maturation took place in a 5% CO _2_ atmosphere. For imaging, we transferred oocytes into a glass-bottom Flourodish (WPI Inc.) containing a single 50-μl droplet of maturation medium and covered with oil. To inhibit fluid flows that cause sample drift, an appropriately sized coverslip was suspended over the interior plastic rim of the glass-bottom Flourodish, effectively confining the sample between horizontal parallel plates (the glass bottom of the dish and the suspended coverslip) separated by ∼1 mm. Before use, culture and imaging medium was pre-equilibrated in a 5% CO _2_ atmosphere at 37°C for at least 2 hours. Oocyte collection and manipulation was carried out on a dissection microscope with a stage heated to 37°C.

### LC-PolScope microscopy and image registration

The LC-PolScope hardware (Cambridge Research Instruments) was mounted on a Nikon TE2000-E microscope equipped with a 0.5× air condenser lens and either 100× numerical aperture (NA) 1.45 oil immersion objective lens or a 60× NA 1.2 water immersion objective lens. We controlled the LC-PolScope hardware and analyzed the images we obtain using the OpenPolScope MicroManager software package (www.openpolscope.org). Before imaging, we manually searched for spindles whose long axes lay in or near the image plane. To confirm spindle orientation, we took a *z* stack of LC-PolScope images with a *z* spacing of 1 μm and visually confirmed that the poles were symmetrical to within ∼2 μm of each other in the direction perpendicular to the image plane; this ensures that $\text{>cosϕ}\equiv \hat{o}\cdot \hat{x}≲0.1$. Since the measured spindle retardance *r*_meas._ scales with the intrinsic retardance *r* (i.e., the retardance that would be measured if the spindle axis were perfectly perpendicular to $\hat{o}$) as *r*_meas_ = *r*cos^2^ϕ, the maximum error between measured and intrinsic spindle retardance is ≲1% in our experiments. Our movies typically consist of ∼500 frames acquired over ∼30 min. During image acquisition, spindles often translated/rotated through several micrometers/degrees. We discarded data from those spindles that rotated or translated out of the image plane, and, for the remainder, used a custom-written image registration algorithm, implemented in the Mathematica programming language, to ensure that all subsequent analysis was performed in the rest frame of the spindle.

Our registration algorithm comprises four steps. First, we manually crop a region of interest (ROI) that includes all positions of the spindle over the course of the entire movie. Second, we apply a Gaussian blur (radius, 3 μm) to each frame of the retardance movie, binarize the image using a threshold of half the maximum intensity, find the center of brightness of the largest contiguous white region (i.e., the spindle), and translate both the retardance image and the corresponding slow axis image such that, in all frames of the movie, the center of the spindle corresponds with the center of the image. Third, we use the slow axis image to rotate the spindle such that its average orientation is parallel to $\hat{x}$, the horizontal axis of the image. Finally, we find the translation that aligns each retardance image to a reference retardance image generated by applying a moving average to the neighboring frames (window width, 10 frames). To do this alignment, we apply a gradient filter to both the target image and the reference image and numerically obtain the translation that maximizes overlap between target and reference images. Once we have found this translation, we apply it to both retardance and slow axis images. None of our later analysis depends sensitively on the details of the image registration; for instance, we can entirely omit the final alignment step without substantially altering any subsequent results. The LC-PolScope images of MII oocytes analyzed in Figs. 1 to 3 were obtained from experiments carried out on four different dates, with oocytes obtained from multiple mice on each date.

### Identifying spindle boundary from time-averaged retardance image; fitting boundary to circle arcs

To identify spindle boundaries, we average the registered retardance images to find the 〈*r*〉*_t_* image (Fig. 1B, middle left), subtract the background value, apply a Gaussian filter (radius, 1.0 μm), and use a gradient filter to locate the boundary (Fig. S4A, top row). We next fit the empirically identified boundary to a set of four circle arcs (Fig. 1C and text S4). To do this, we plot the empirical boundary **b**(ϕ) in polar coordinates in the *xy* plane and identify the portions of the boundary corresponding to the convex and concave parts of the pole-indented tactoid. We find the circle arcs that best fit the convex portions, and thus the spindle belt width 2*R*_0_ and virtual pole positions (±*L*_0_,0). Finally, we find the radius *r*_0_ of the circles, centered on (±*L*_0_,0), that best fit the concave portions of the spindle surface (Fig. 1B, bottom left, and Figs. S4A and S5).

### Averaging best-fit boundaries of different spindles

To average the fitted boundaries of *n* spindles, we first rescale the fit parameters $R_{0}^{i}$ and $r_{0}^{i}$ of spindle *i* by the corresponding virtual pole half-spacing $L_{0}^{i}$ and average these quantities to define the average normalized pole-indented tactoid (Fig. 1, D and G).

### Averaging angle fields

Microtubule orientation must be averaged in a way that respects its nematic symmetry: Since θ = 0 and θ = π are equivalent, their average is not π/2 but 0 (or π). To do this, we average a set of orientations ${\left\{\theta_{j}\right\}}_{j=1}^{N}$ using the formula

$$\langle \theta \rangle \equiv \frac{1}{2}\text{arg}\sum_{j=1}^{N}‍e^{2i\theta_{j}}$$

To obtain the angle slices through the spindle shown in Fig. 1H, we transform the data from each individual spindle into the first quadrant before averaging over all spindles. To do this, we take advantage of the expected odd symmetry of the angle field under reflection about the $\hat{x}$ and $\hat{y}$ axes, θ(−*x*, *y*) = θ(*x*, −*y*) = −θ(*x*, *y*). For a given point {*x*_1_, *y*_1_} in the first quadrant, the associated angle is therefore [θ(*x*_1_, *y*_1_) − θ(*x*_1_, −*y*_1_) − θ(−*x*_1_, *y*_1_) + θ(−*x*_1_, −*y*_1_)]/4.

### Calculation of experimental correlation functions

We calculate correlation functions in ROIs, boxes with side length λ_0_ = 8 μm placed at the center of the spindle (Fig. 2A). In these boxes, ∣〈θ〉*_t_* ∣ ≲ 15^∘^ and so δ*n_y_* = δθ to within ∼1% accuracy (Fig. 2B). For each point in the ROI, we directly calculate the fluctuation δ*n_y_*(**r**, *t*) ≈ θ(**r**, *t*) − θ_arcs_(**r**), where θ_arcs_(**r**) is obtained from the family of circle arcs intersecting at points (±*L*_0_,0), found from fitting the boundary of the time-averaged retardance image 〈*r*〉*_t_* (Fig. 1B and Fig. S5). We use Mathematica’s inbuilt Fourier[] function to approximate the (2 + 1)D Fourier transform of the fluctuations, ${\tilde{\delta n}}_{y}\left(q,\omega \right)=\int ‍dq\mathrm{d\omega}\;\delta n_{y}\left(r,t\right)e^{-i\left(\omega t+q\cdot r\right)}$, inside the ROI, and thus the spatiotemporal and equal-time correlation functions *c*_nn_(**q**, ω) and *s*_nn_(**q**, ω) (text S6)

$$c_{\text{nn}}\left(q,\omega \right)=\frac{1}{\tau_{0}\lambda_{0}^{2}}{\tilde{\delta n}}_{y}{\left(q,\omega \right)}^{*}{\tilde{\delta n}}_{y}\left(q,\omega \right);\;\;s_{\text{nn}}\left(q\right)=\frac{1}{2\pi }\int ‍c_{\text{nn}}\left(q,\omega \right)\mathrm{d\omega}$$

where τ_0_ is the total time of the movie being analyzed and the integral in the definition of *s*_nn_(**q**) is taken over all available angular frequencies ω. To minimize boundary artifacts, we impose “mirror padding” in all dimensions before computing Fourier transforms (*47*). To avoid artificial correlations induced by the finite diffraction limit of the imaging system, we discard data points for which ∣**q**∣ > 2π/λ_im_, where λ_im_ = 530 nm is the microscope illumination wavelength.

### Analyzing correlations between retardance and orientational fluctuations

To analyze the real-space structure of retardance variations and orientational fluctuations, we use a local adaptive binarization algorithm. If the pixel at position {*i*, *j*} has intensity *I*(*i*, *j*), the binarization algorithm transforms *I*(*i*, *j*) according to the function

$$I_{\text{loc}.\text{bin}.}\left(i,j\right)=\{\begin{array}{c} 0\;\text{if I}\left(i,j\right)<\langle I\left(m,n\right)\rangle \\ 1\;\text{if I}\left(i,j\right)\geq \langle I\left(m,n\right)\rangle \end{array}$$

where the average is taken over all pixels {*m*, *n*} that lie within 2 μm of {*i*, *j*}.

### Confocal microscopy and chromosome identification

Confocal stacks such as those shown in Fig. 3 (A and B) were acquired on an inverted Leica SP5 microscope, with solid-state 488- and 633-nm lasers, using a 63× 1.4 NA oil immersion objective lens. We acquired stacks of the entire spindle with separation 1 to 2 μm along the optical axis and pixel size ≈100 nm in the image plane. The expected resolution in the chromosome channel λ_res_ is given by the formula λ_res_ ≈ 488 nm/(2NA) ≈ 174 nm. To analyze the chromosome configuration in the metaphase plate (Figs. 3B and 4A), we selected confocal images of spindles whose long axes $\hat{x}$ were oriented within ≈30° of the optical axis $\hat{o}$. To find an image of the metaphase plate, we numerically rotate/reslice the confocal stack to ensure that the best-fit plane to the chromosome channel is the *x* = 0 plane (Fig. 3B). To identify chromosome/void edges in the metaphase plate, we binarize the *x* = 0 image by first applying a Gaussian blur of width 2 μm, and a local adaptive binarization algorithm of radius 1.5 μm, and finally an erosion filter with kernel width 0.4. None of the results we present depends sensitively on our specific choice of these or other image analysis parameters. Our procedure identifies *n*_chr_ white objects on a black background; we keep for further analysis those datasets (11 of a total of 20 spindles originally imaged) for which *n*_chr_ = 19 or 20 and discard the others. If we instead include all datasets, this does not substantially change any of the results we present. In spindles where 19 ≤ *n*_chr_ ≤ 20, chromosomes sections have an average area of 1.45 ± 0.03 μm^2^, and chromosomes occupy ∼20% of the area of the metaphase plate, comparable to the ∼15% “missing” area identified by LC-PolScope (Fig. 3E and Fig. S10). The chromosome images analyzed in Fig. 4 were taken from experiments carried out on three different dates, with oocytes obtained from multiple mice on each date.

### Live cell staining and mRNA microinjection

To image microtubules, we added the live-cell stain SiR-tubulin (Cytoskeleton Inc.) to the culture medium at least 30 min before imaging, at a concentration of 0.5 to 1 μM. To minimize the exposure of the oocytes to dimethyl sulfoxide (DMSO), we performed intermediate dilutions into aliquots of maturation medium rather than DMSO. We did this immediately before adding the stain to the culture medium. To simultaneously image microtubules and DNA, we synthesized mRNA coding for the fluorescently tagged histone protein H2B-EGFP using the HiScribe T7 Arca kit (New England Biolabs) according to the manufacturer’s instructions. Using a previously described technique, we injected mRNA solution, diluted to a concentration of ∼1 μg/μl into GV oocytes at volumes approximately equal to 1% of the oocyte volume (*48*). For the injections, we used the Narishige micromanipulation system (Narishige International US Inc.) for oocyte manipulation, a PV850 microinjector (World Precision Instruments) for mRNA delivery, and an IE-251A intracellular electrometer (Warner Instruments) for delivering the capacitive pulse that helps the oocytes survive the injection. We performed injections on a Nikon TE2000-E microscope mounted with a 10× objective lens and equipped with crossed polarizers to increase contrast. Injections took place at room temperature in medium containing the meiotic inhibitor cocktail. After injecting mRNA, we released the GV oocytes from meiotic arrest and allowed them to mature as usual.

To image microtubules, we added the live-cell stain SiR-tubulin (Cytoskeleton Inc.) to the culture medium at least 30 min before imaging, at a concentration of 0.5 to 1 μM. To minimize the exposure of the oocytes to dimethyl sulfoxide (DMSO), we performed intermediate dilutions into aliquots of maturation medium rather than DMSO. We did this immediately before adding the stain to the culture medium. To simultaneously image microtubules and DNA, we synthesized mRNA coding for the fluorescently tagged histone protein H2B-EGFP using the HiScribe T7 Arca kit (New England Biolabs) according to the manufacturer’s instructions. Using a previously described technique, we injected mRNA solution, diluted to a concentration of ∼1 μg/μl into GV oocytes at volumes approximately equal to 1% of the oocyte volume (*48*). For the injections, we used the Narishige micromanipulation system (Narishige International US Inc.) for oocyte manipulation, a PV850 microinjector (World Precision Instruments) for mRNA delivery, and an IE-251A intracellular electrometer (Warner Instruments) for delivering the capacitive pulse that helps the oocytes survive the injection. We performed injections on a Nikon TE2000-E microscope mounted with a 10× objective lens and equipped with crossed polarizers to increase contrast. Injections took place at room temperature in medium containing the meiotic inhibitor cocktail. After injecting mRNA, we released the GV oocytes from meiotic arrest and allowed them to mature as usual.

## Supplementary Materials

### This PDF file includes:

Supplementary Text Tables S1 and S2 Figs. S1 to S16 References
