# Supplementary material for: Long-range repulsion between chromosomes in mammalian oocyte spindles
Source: Sci Adv. 2024 Sep 25;10(39):eadq7540. doi: 10.1126/sciadv.adq7540 (PMC11423871; doi:10.1126/sciadv.adq7540)
Supplement: Supplementary file 1 — Supplementary Text Tables S1 and S2 Figs. S1 to S16 References [file sciadv.adq7540_sm.pdf]

Science Advances
AAAS

Supplementary Materials for

Long-range repulsion between chromosomes in mammalian oocyte spindles

Colm P. Kelleher *et al.*

Corresponding author: Colm P. Kelleher, colm.p.kelleher@gmail.com

Sci. Adv. **10**, eadq7540 (2024)
DOI: 10.1126/sciadv.adq7540

This PDF file includes:

Supplementary Text
Tables S1 and S2
Figs. S1 to S16
References

Supplemental Text

1 Formation and Lifetimes of MI & MII Spindles in Mouse Oocytes

As described in Materials & Methods, we extract germinal-vesicle-stage (GV-stage) oocytes from mouse ovaries and mature them *in vitro*. LC-PolScope observations indicate that, after completion of anaphase I, MII oocytes spindles remain in steady state for at least 12 hours (Fig. S1).

2 Retardance Images Behave As If Light Were Collimated as It Passes Through Spindle

In this work, we treat all LC-PolScope data as if it represented a simple projection over the optical axis $\hat{o}$ (Main Text Eqns. 1). This is equivalent to modeling the light passing through the spindle as collimated, i.e. we assume that the optical section depth of the LC-PolScope is much larger than the spindle diameter. This assumption is consistent with previous measurements of invertebrate oocytes in a quantitative polarization microscopy system similar to the LC-PolScope (*22*). In that work, it was shown that measured retardance values do not depend on the details of the optical train, in particular on the numerical apertures (NAs) of the objective and condenser lenses, consistent with the assumption of collimated light. However, we used immersion objectives with significantly higher NAs than reference (*22*), and, to our knowledge,

there is not a simple way to theoretically estimate the optical section depth of "semi-coherent" microscopy like LC-PolScope (*51*). To experimentally check whether our results depend on the details of the optical train, we measured the retardance profiles of MI and MII spindles with three different objectives, whose NAs varied from 0.10 (magnification 4x) to 1.45 (magnification 100x). Consistent with the results of the previous study, we found that our retardance measurements do not depend strongly on the NA or magnification of the objective lens used, and conclude that, in all cases, transmitted light behaves as though it were approximately collimated (Fig. S2). In all LC-PolScope experiments, we used a condenser lens with NA 0.52, with the condenser iris in the half-closed position such that the effective condenser NA ≈ 0.25. We do not expect the condenser NA to significantly affect the lateral resolution of the microscope (*52*).

3 Calculation of Microtubule Cross-Sectional Density from Observed Retardance

3.1 Retardance of Microtubule Arrays Aligned Perpendicular to the Optical Axis

Microtubules exhibit "form birefringence"; that is, their birefringence arises predominantly from their rod-like shape and their high refractive index relative to the surrounding medium, rather than "stress birefringence" (arising from anisotropic mechanical strain) or "intrinsic birefringence" (arising from anisotropic distribution of oriented chemical bonds) (*22*). Here, we construct a model, based on Wiener's equation for form birefringence (*53*), that relates the observed retardance of a sample of microtubules coherently aligned in a direction $\hat{\mathbf{x}}$ perpendicular to the optical axis $\hat{\mathbf{o}}$ with the average 2D cross-sectional density ρ_0 of the microtubules in the plane perpendicular to $\hat{\mathbf{x}}$. By definition, the retardance of a sample is proportional to its thickness T and the refractive index mismatch $\Delta n = n_{\text{slow}} - n_{\text{fast}}$ between light polarized along the

fast and slow optical axes,

$$r = T\Delta n.$$

For materials like microtubules, whose birefringence arises from their rod-like shape, Δn is related to the volume fraction f and refractive index n_{MT} of the microtubules, as well as the refractive index n_{Cyto} of the surrounding cytoplasm,

$$\Delta n = f \frac{(n_{\text{Cyto}}^2 - n_{\text{MT}}^2)^2}{2n_{\text{Cyto}}(n_{\text{Cyto}}^2 + n_{\text{MT}}^2)}.$$

This expression assumes $f \ll 1$; this assumption is generally appropriate for spindle microtubules (*54*). For a bulk material composed of aligned rods, it is the also case that

$$f = \pi \left(\frac{d_{MT}}{2}\right)^2 \rho_0,$$

where d_{MT} is the diameter of a single rod. Putting these equations together, we obtain an equation relating r and ρ_0,

$$\rho_0 = \frac{r}{TA_0}, \tag{S1}$$

where the so-called retardance area A_0 characterizes the contribution of a single microtubule to the measured retardance,

$$A_0 \equiv \left(\frac{\pi d_{MT}^2}{4}\right) \frac{(n_{\text{Cyto}}^2 - n_{\text{MT}}^2)^2}{2n_{\text{Cyto}}(n_{\text{Cyto}}^2 + n_{\text{MT}}^2)}.$$

The microtubule-related quantities d_{MT} and n_{MT} have been previously measured, and should not depend strongly on cell type: $d_{MT} \approx 24$ nm and $n_{\text{MT}} \approx 1.512$ (*22, 55*). To our knowledge, the refractive index of mouse oocyte cytoplasm has not been measured. For other metazoan cell types, n_{Cyto} typically lies in the range 1.35-1.40 (*22, 56*). Assuming that $n_{\text{Cyto}} = 1.375 \pm 0.025$, we obtain $A_0 = (6.2 \pm 1.8)$ nm^2, consistent with previous experimental measurements of this quantity *in vitro*, which yield $A_0 \approx 7.5$ nm^2. (*23*). Using the measured values of microtubule cross-sectional density given in the Main Text, we find $f \approx 0.04$, consistent with the assumption $f \ll 1$ used in the above derivation.

3.2 Average Microtubule Cross-Sectional Density in MI and MII Spindles

To estimate the average microtubule cross-sectional density $\langle\rho\rangle$ (Fig. S1(c)), we first calculate the average retardance in a $8\,\mu$m $\times$ $8\,\mu$m box in the center of the spindle, and take the spindle diameter as the sample thickness, $T \approx 2R_0$, and directly apply Eqn. S1.

3.3 Predicted Retardance Profile of Oocyte Spindle

To measure the dependence of microtubule density on position along the spindle axis (Main Text Fig. 3 (d & e)), we construct a more detailed model which takes into account the fact that spindles do not have uniform thickness, but are well-described as surfaces of revolution with axes of revolution $\hat{\mathbf{x}}$,

$$\mathbf{R}(x, \phi) = \{x, R(x)\cos\phi, R(x)\sin\phi\},$$

where $R(x)$ is the spindle radius at postion x along its long axis, and $\tan\phi \equiv y/z$ is the cylindrical polar coordinate relative to the $\hat{\mathbf{x}}$-axis. Assuming collimated light and that the spindle is centered at the origin, the sample thickness corresponding to a specific position $\{x, y\}$ in the LC-PolScope image plane is given by the formula $T(x, y) = 2(R(x)^2 - y^2)^{1/2}$, and Eqn. S1 may be rewritten as

$$r(x, y) = 2\langle\rho\rangle_z (R(x)^2 - y^2)^{1/2}, \tag{S2}$$

where $\langle\rho\rangle_z$ is the cross-sectional density averaged over the optical path through the spindle (Fig. S3). To find the average cross-sectional density in the retardance slice $x = x_i$, we treat density $\rho_x(x_i)$ and spindle radius $R(x_i)$ as independent fit parameters in the equation

$$r(x_i, y) = 2\rho_x(x_i)(R(x_i)^2 - y^2)^{1/2},$$

where the density profile $\rho_x(x)$ along the spindle axis may be interpreted as the 3D density profile averaged over y and z, $\rho_x(x) \equiv \langle\rho(x, y, z)\rangle_{y,z}$. For time-averaged retardance images, we consider $\rho_x(x)$ to be averaged over time as well, $\rho_x(x) \equiv \langle\rho(x, y, z, t)\rangle_{y,z,t}$.

4 Fitting Spindle Boundary to Circle Arcs

4.1 Rationale for Fitting Spindle Boundary to Circle Arcs

In the analysis presented in Fig. 1 of the Main Text, we fit empirically observed spindle boundaries, obtained from time-averaged LC-PolScope retardance images, to a set of circle arcs fully determined by the three geometrical parameters L_0, R_0, and r_0 (Main Text Fig. 1 (b & c)). We chose this family of shapes for two reasons: first, a very similar shape has been shown to arise from a mechanical model in which molecular-motor-driven contractile forces balance stresses from nematic elasticity at the spindle boundary (*17*). Second, the circle arc family provides better fits to the empirically measured spinde boundaries (Materials & Methods, Fig. S4) than other curve families we have tested, for example ellipses or rectangles. The utility of the circle arc family is particularly evident when we fit confocal slices that contain the spindle long axis; in these micrographs, the concave geometry of many (but not all) polar caps is more visually evident than in LC-PolScope retardance images (Figs. S5(a&b)). This discrepancy is presumably due to the fact that the $\hat{\mathbf{z}}$-projection implicit in the LC-PolScope measurement obscures the true shape of the cross-section.

4.2 Shape Parameters from Confocal Slices Vs Shape Parameters from LC-PolScope Retardance Images

The shape parameters (L_0, R_0, and r_0) found by fitting confocal sections are systematically $\sim$10% greater than those found from analysis of time-averaged LC-PolScope retardance images (Fig. S5 (c & d)), presumably due to errors associated with the fact that the long axes of spindles imaged with the LC-PolScope are not perfectly perpendicular to the optical axis (Materials & Methods) and systematic errors in identifying the spindle boundary from a retardance images (Materials & Methods). We consider the parameters obtained from fitting the confocal images to more accurately characterize the true shapes of living MII spindles.

5 2D Circle Arcs Model Is a Good Approximation of $\hat{\mathbf{z}}$-Projected 3D Circle Arcs Model

The orientation fields we obtain from LC-PolScope are not the full 3D orientation fields; rather, they are those fields projected over an axis, $\hat{\mathbf{z}}$, that is perpendicular to the spindle long axis $\hat{\mathbf{x}}$ (Main Text Fig. 1). Therefore, previously derived predictions for 3D nematic tactoids, e.g. the circle arcs model (*29*), do not directly apply to our data (Fig. S6). Here, we estimate the error incurred by approximating the angle fields derived from projections of 3D circle arcs with the 2D circle arcs model. To do this, we construct the 3D nematic field for the nematic tactoid that is uniquely specified by the defect half-spacing L_0 and the radius of curvature R_c of the tactoid boundary (Fig. S6(e); (*30*)). We then calculate the projection of that nematic field into 2D along an axis perpendicular to the tactoid long axis, and compare it to the field generated by the 2D family of circle arcs generated with the same defect spacing and radius of curvature. Our comparison includes both the fields in the central region of the tactoid (the region that describes oocyte spindles very well; Main Text Fig. 1), as well as fields in the polar regions (absent in oocyte spindles). The error we calculate here can therefore be considered as an upper bound on the error associated with comparing the measured $\hat{\mathbf{z}}$-projection of the 3D orientation field with a 2D circle arcs model in mouse oocyte spindles. In the geometry relevant to mouse oocyte spindles ($R_c/L_0 \approx 1.5$), the error introduced by using a 2D circle arcs model to describe projected 3D data is around 1% (Fig. S6(f)).

6 Orientational Correlation Functions

6.1 Active Nematic Model for Equation of Motion for δN (Real & Fourier Space)

In equilibrium field theories, the equipartition theorem can be applied to an appropriately diagonalized expression for free energy to calculate the spectrum of fluctuations (*28*). In non-equilibrium systems like the spindle, we can recover an analogous result by constructing an equation of motion and applying the principles of fluctuating hydrodynamics (*57*). Quite generally, the director field of an active nematic obeys an equation of motion derived from local torque balance (*13, 58*)

$$\gamma\big(\frac{\partial \hat{N}_i}{\partial t} + v_j \nabla_j \hat{N}_i - M_{ij}\hat{N}_j\big) = -h_i + \gamma S_i. \tag{S3}$$

Here, $\mathbf{v}$ is the hydrodynamic velocity field of microtubules plus surrounding fluid, the tensor $\underline{\underline{M}} = 1/2(\nabla\mathbf{v} - (\nabla\mathbf{v})^T) + \lambda/2(\nabla\mathbf{v} + (\nabla\mathbf{v})^T)$ describes the flow alignment of microtubules, γ is a rotational viscosity, λ dictates how microtubules orient in shear flows, S_i is a noise term whose form we will specify later. Using the one-constant approximation $k \equiv k_{\text{bend}} = k_{\text{twist}} = k_{\text{splay}}$, the molecular field h_i is given by $h_i = k\nabla^2 n_i$ (*28*). In general, Eqns. S3 must be supplemented by the constraint that the director is a unit vector, $|\hat{\mathbf{N}}| = 1$, and a stress-balance equation which sets $\mathbf{v}$. For an active nematic at low Reynolds number, the stress balance equation may be written

$$\underline{\underline{\sigma}}^r + \underline{\underline{\sigma}}^v + \underline{\underline{\sigma}}^a = 0,$$

where $\underline{\underline{\sigma}}^r$, $\underline{\underline{\sigma}}^v$, and $\underline{\underline{\sigma}}^a$ are the reactive, viscous, and active contributions to the total stress, given by

$$\sigma^r_{ij} = p\,\delta_{ij} - \frac{\lambda}{2}(n_i h_i + n_j h_j);$$
$$\sigma^v_{ij} = -\eta\,\partial_j(\partial_i v_j + \partial_j v_i);$$
$$\sigma^a_{ij} = -\zeta n_i n_j,$$

where p is the hydrostatic pressure, η is the dynamic viscosity of the composite fluid, and ζ is the active stress density. However, it has previously been argued (*13*) that the flows in steady-state *in vitro* assembled Xenopus egg extract spindles are so small that the terms involving $\mathbf{v}$ in Eqn. S3 may be neglected. In that case, the equation of motion for small fluctuations $\delta\hat{\mathbf{N}}$ about the director $\hat{\mathbf{x}}$ becomes the diffusion equation with non-conservative noise,

$$\frac{\partial \delta N_j}{\partial t} = -K\nabla^2 \delta N_j + S_j, \qquad \text{(S4)}$$

where δN_j, $j \in \{y, z\}$, are the components of the fluctuation perpendicular to the director, and $K \equiv k/\gamma$ is the nematic diffusivity, which has units of diffusion constant. Taking the Fourier transform of this expression, we get

$$-i\omega\delta\tilde{N}_j = K\mathbf{Q}^2\delta\tilde{N}_j + \tilde{S}_j(\mathbf{Q}, \omega),$$

where $\mathbf{Q} = \{q_x, q_y, q_z\}$ and ω are the wave-vector and angular frequency, and the Fourier transform is defined as

$$\tilde{f}(\mathbf{Q}, \omega) \equiv FT\{f(\mathbf{R}, t)\} \equiv \int \mathrm{d}\mathbf{Q}\,\mathrm{d}\omega\, e^{-i(\mathbf{Q}\cdot\mathbf{R}+\omega t)} f(\mathbf{R}, t),$$

where $f(\mathbf{R}, t)$ is an arbitrary function of space and time.

6.2 Theoretically Predicted Fourier Space Correlation Functions in (3+1)D

To explicitly calculate correlation functions from the equation of motion S4, we assume Gaussian-distributed noise. In real space, $S_j(\mathbf{R},t)$ is fully characterized by the equations

$$\langle S_j(\mathbf{R},t)\rangle = 0, \qquad \langle S_i(\mathbf{R},t)S_j(\mathbf{R}',t')\rangle = S_0^2\,\delta(\mathbf{R}-\mathbf{R}')\delta(t-t')\delta_{ij},$$

where S_0^2 is the noise amplitude. These relations are equivalent to the Fourier space expressions

$$\langle \tilde{S}_j(\mathbf{Q},\omega)\rangle = 0, \qquad \langle \tilde{S}_i(\mathbf{Q}_1,\omega_1)\tilde{S}_j(\mathbf{Q}_2,\omega_2)\rangle = S_0^2\,\delta(\mathbf{Q}_1+\mathbf{Q}_2)\delta(\omega_1+\omega_2)\delta_{ij}.$$

Using these expressions, we calculate the full spatiotemporal correlation function for $\delta\tilde{N}_y$,

$$C_{\text{nn}}(\mathbf{Q},\omega) \equiv \frac{1}{V_{3+1}}\delta\tilde{N}_y(\mathbf{Q},\omega)^*\delta\tilde{N}_y(\mathbf{Q},\omega) = \frac{S_0^2}{\omega^2+K^2\mathbf{Q}^4}, \tag{S5}$$

where V_{3+1} is the (3+1)D system volume. The equal time correlation function is given by evaluating the inverse Fourier transform (in the time domain only) of $C_{\text{nn}}(\mathbf{Q},\omega)$ at $t=0$,

$$S_{\text{nn}}(\mathbf{Q}) = \frac{1}{2\pi}\int_{-\infty}^{\infty} C_{\text{nn}}(\mathbf{Q},\omega)d\omega = \frac{S_0^2}{2K\mathbf{Q}^2}. \tag{S6}$$

6.3 LC-PolScope Measurements, Projection Slice Theorem, Correlation Functions in (2+1)D

The LC-PolScope does not allow measurement of the full 3D director $\hat{\mathbf{N}}(\mathbf{R})$. Rather, we measure the orientation field projected over the optical axis $\hat{\mathbf{z}} = \hat{\mathbf{o}}$ (Main Text Eqns. 1 & Fig. 1(a)). According to the projection-slice theorem of Fourier analysis, this is equivalent to taking a long wavelength limit in the $\hat{\mathbf{z}}$ direction, i.e. $q_z \to 0$ (*59*). In operator notation,

$$S_{q_z=0}FT_{xyz} = FT_{xy}P_z,$$

where $S_{q_z=0}$ is the slice operator that sets $q_z \to 0$ in its argument function, FT_{xy} and FT_{xyz} are 2D and 3D spatial Fourier transforms, P_z is the projection operator over z, $P_z\{f(x,y,z)\} =$

$\int f(x, y, z)\mathrm{d}z$, and we have suppressed the time dimension for convenience. With this notation,

$$\tilde{\delta N}_y(q_x, q_y, q_z \to 0) = F_{xy} P_z \delta N_y \approx F_{xy} \int_T \delta N_y \mathrm{d}z \approx T \tilde{\delta n}_y,$$

where T is the sample thickness along $\hat{\mathbf{z}}$. In the above expression, the first approximation enters because of the finite sample thickness, and the second arises because $\hat{n}$ is a normalized projection rather than an absolute projection, see Eqns. 1 of the Main Text. (The last relation is accurate to first order in δN_j, however.) Applying the limit $q_z \to 0$ to equations S5 and S6 therefore yields

$$c_{\mathrm{nn}}(q_x, q_y, \omega) \equiv \frac{1}{V_{2+1}} \tilde{\delta n}_y(\mathbf{q}, \omega)^* \tilde{\delta n}_y(\mathbf{q}, \omega) = \frac{s_0^2}{\omega^2 + K^2(q_x^2 + q_y^2)^2}; \qquad s_{\mathrm{nn}}(q_x, q_y) = \frac{s_0^2}{2K(q_x^2 + q_y^2)}, \tag{S7}$$

where $V_{2+1} = V_{3+1}/T$ is the (2+1)D system volume, and $s_0^2 \equiv S_0^2/T$. As shown in (*13*), In the long wavelength limit in the direction along the spindle axis, $q_x \to 0$, s_{nn} takes a particularly simple inverse-square power law form,

$$s_{\mathrm{nn}}(0, q_y) = \frac{s_0^2}{2Kq_y^2}. \tag{S8}$$

6.4 Comparison of Experimental Correlation Functions with Theoretical Predictions; Estimate of Position of Peak in $s_{\mathbf{nn}}(q_y)$

Fits of Eqns. S7 and S8 to the correlation functions calculated from LC-PolScope data are shown in Fig. S7(a & b). We use these fits to estimate the location q_y^* of the anomalous peak in $s_{\mathrm{nn}}(q_0, q_y)$ as follows: we first calculate, in log space, the residuals corresponding to each fit (Fig. S7(c)). We then use an ad-hoc fit (a polynomial of degree 10) to obtain a continuous approximation of the residual as a function of q_y, and use Newton's method to identify the value of q_y corresponding to its maximum value. The value of q_y^* we obtain using this method is not sensitive to paramaters such as the number of bins used to calculate the average experimental s_{nn} or the specific form of the ad-hoc approximation to the residual curve. To obtain the uncertainty

in q_y^* reported in the Main Text, we perform empirical bootstrapping over single-spindle data sets: we first randomly select 50% of the single-spindle $s_{\rm nn}$ curves (i.e. 50% of the faint colored curves in Fig. S7(a)) and obtain a value of q_y^* using only that data. We then repeat this procedure 100 times, and take the standard deviation of the resulting distribution of q_y^* values to be the uncertainty in q_y^*.

6.5 Regularly Spaced Voids in a q^{-2}-Correlated Background Generate a Peak in $s_{\rm nn}(q)$: Simulations

To understand in greater detail how voids in the microtubule network lead to the observed peak in $s_{\rm nn}(q_0, q_y)$ (Main Text Fig. 2(c)), we performed a series of simulations (Fig. S8(a-c)). To model fluctuations in the microtubule network in the absence of voids, we used a forward time-centered space (FTCS) scheme to numerically integrate a dimensionless (1+1)-D version of Eqn. S4 for fluctuations in a contiguous active nematic, i.e. the 1D diffusion equation with non-conservative Gaussian noise,

$$\frac{\partial \psi}{\partial t} = -\frac{\partial^2 \psi}{\partial x^2} + S(x,t), \tag{S9}$$

where $\psi(x,t)$ is the fluctuating field and the scalar noise $S(x,t)$ is defined by the equations $\langle S(x,t)\rangle = 0$, $\langle S(\mathbf{x},t)S(\mathbf{x}',t')\rangle = \delta(x-x')\delta(t-t')$. Choosing a time-step that satisfies the stability criterion $\Delta t \leq \Delta x^2/2$, we integrate this equation on the spatio-temporal domain $[0,1]\times[0,2]$; this choice of domain allows relaxation of the longest-wavelength modes. We impose Dirichlet boundary conditions $\psi(0,t) = \psi(1,t) = 0$ for all t, and initialize the simulation with $\psi(x,0) = 0$ for all x. In all subsequent analysis, we consider only the values of $\psi(x,t)$ for $t \in [1,2]$, for which the simulation samples the steady-state distribution of field configurations. A snapshot of the fluctuating field at $t = 1$ is shown in Fig. S8(a), as is the equal-time correlation function $S_{\psi\psi}(x)$, averaged over all $t \in [1,2]$; the latter plot decays as q^{-2} as predicted by Eqn. S8.

To model the effect of enhanced fluctuations in regularly spaced voids, we construct another field $\chi(x,t)$ by replacing the simulated values of $\psi(x,t)$ in N periodically spaced intervals ("patches"), whose centers $c_i = \{i/(N+1)\}_{i=1,N}$ are separated by wavelength $\lambda_{\text{int}} = 1/(N+1)$, and which have width $w_0 < \lambda_{\text{int}}$. In those intervals, $\chi(x,t)$ is doubled,

$$\chi(x,t) = \begin{cases} 2\psi(x,t) & x \in (c_i - \frac{w_0}{2}, c_i + \frac{w_0}{2}) \text{ for any } i, \\ \psi(x,t) & \text{otherwise.} \end{cases}$$

Having constructed a real-space model of a field with increased fluctuations in regularly spaced patches, we next compute its equal-time correlation function $S_{\chi\chi}(q)$ (Fig. S8(b)). For appropriate choice of the parameters N and w_0, $S_{\chi\chi}(q)$ displays a peak at $2\pi/\lambda_{\text{int}}$ superimposed on a q^{-2} decay that predominates at both higher and lower q-values, similar to the form of the $S_{\text{nn}}(q_0, q_y)$ plot calculated from experimental observations (Main Text Fig. 2(c)). A similar result can be recovered if, instead of doubling the field in the patches, the correlated fluctuations are replaced by spatially uncorrelated Gaussian white noise; this implies that the peak in $S_{\chi\chi}$ is not sensitive to the specific behavior of $\chi(x,t)$ in the patches (Fig. S8(c)).

6.6 Regularly Spaced Voids in a q^{-2}-Correlated Background Generate a Peak in $s_{\text{nn}}(q)$: Analytical Calculation

By using the convolution theorem of Fourier analysis, the emergence of peaks in $S_{\text{nn}}(q_0, q_y)$ can also be understood from a theoretical perspective. Let $\psi(x)$ be a real-valued function with the power spectrum $|\tilde{\psi}(q)|^2 = |A_0|^2 q^{-2}$. (For functions of both space and time, the time-averaged spatial power spectrum is identical to the equal-time correlation function $S_{\psi\psi}(q)$ apart from a constant normalization factor.) This form of $|\tilde{\psi}(q)|^2$ implies that $\tilde{\psi}(q) = A_0 q^{-1}$, where A_0 may be complex-valued. We represent periodic voids by multiplying $\psi(x)$ by a sinusoidal function with minimum zero and maximum 1,

$$\chi(x) \equiv \psi(x)(1 - \sin kx)/2,$$

where k is the angular wavenumber associated with the spatial period of the sinusoid. Taking the Fourier transform of $\chi(x)$ and applying the convolution theorem yields

$$\tilde{\chi}(q) = \frac{1}{2}\Big(\tilde{\psi}(q) - FT\{\psi(x)\sin kx\}\Big) = \frac{1}{2}\Big(\tilde{\psi}(q) - \frac{1}{2i}(\tilde{\psi}(q-k) - \tilde{\psi}(q+k))\Big).$$

From this expression we can directly calculate the power spectrum $|\tilde{\chi}(q)|^2$,

$$|\tilde{\chi}(q)|^2 = \frac{1}{4}\Big(|\tilde{\psi}(q)|^2 + \frac{1}{4}(|\tilde{\psi}(q-k)|^2 + 2|\tilde{\psi}(q-k)||\tilde{\psi}(q+k)| + |\tilde{\psi}(q+k)|^2)\Big) = \frac{|A_0|^2}{4}\frac{q^4 + (q^2-k^2)^2}{q^2(q-k)^2(q+k)^2},$$

which has a divergence at $q = k$ and is proportional to q^{-2} for both $q \gg k$ and $q \ll k$, as expected (Fig. S8(d-e)).

7 Correlations Between Retardance and Fluctuation Magnitude, Corrected for Varying Spindle Thickness

In Fig. 2 of the Main Text and associated discussion, we demonstrated that the time-averaged retardance $\langle r \rangle_t$ at each pixel is negatively correlated with the time-averaged magnitude of orientational fluctuations $\langle |\delta\theta| \rangle_t$. However, without further analysis, it is possible that this is an artifact caused by the fact that retardance is proportional to sample thickness: For an approximately axisymmetric shape like the spindle, with its rotational axis in the image plane, the sample thickness will be largest at the axis and decay to zero at the boundaries. Thus, if the spindle were uniform density, observed retardance would be highest along the long axis of the spindle $y = 0$ and lowest at the spindle boundary (Eqn. S2; Main Text Fig. 3(d)). At the same time, because of interactions between microtubules and the spindle boundary, orientational fluctuations could in principle be either enhanced or suppressed near the edge of the spindle, which might lead to (positive or negative) correlations between retardance and orientaiontal fluctuations that are not due to a direct relationship between them, but instead arise from the fact that that both depend on distance from the boundary. To remove this potential artifact, we constructed the

corrected retardance

$$\langle r' \rangle_t(x, y) \equiv \frac{\langle r \rangle_t(x, y)}{h(x, y)},$$

where $h(x, y)$ is the relative z-thickness at position (x, y) in the spindle (Fig. S9(a)). To estimate $h(x, y)$, we first identify the y-coordinates $y_1(x)$ and $y_2(x)$ of the spindle boundary at a given position x along the spindle long axis. For that value of x, $h(x, y)$ is then given by the equation of the arc of an ellipse with maximum height unity and values of zero at each boundary,

$$h(x, y) = \sqrt{1 - \left(\frac{-2y + y_1 + y_2}{y_1 - y_2}\right)^2}.$$

To avoid the singular values of $\langle r' \rangle_t(x, y)$ that appear (by construction) at the spindle boundary, we construct a tighter mask by removing $1\,\mu$m from the original mask. After making these modifications, repeating the analysis shown in Fig. 2 of the Main Text with $\langle r' \rangle_t(x, y)$ instead of $\langle r \rangle_t(x, y)$ yields results that are identical within experimental uncertainty (Fig. S9(b-e); Table S1).

	Analysis using $\langle r \rangle_t(x, y)$	Analysis using $\langle r' \rangle_t(x, y)$
fraction of oocytes with negative slope of $\langle \lvert\delta\theta\rvert \rangle_t$ vs (adjusted) retardance	15/16	15/16
average slope of $\langle \lvert\delta\theta\rvert \rangle_t$ vs (adjusted) retardance (mean $\pm$ SE, rad/nm^{-1})	-0.009 ± 0.003	-0.010 ± 0.002
average value of $\langle \lvert\delta\theta\rvert \rangle_{\text{low } r} /\ \langle \lvert\delta\theta\rvert \rangle_{\text{high } r}$ (mean $\pm$ SE)	1.07 ± 0.02	1.08 ± 0.03

Table S1: Orientational fluctuation vs retardance: comparison of parameter values obtained using time-averaged retardance $\langle r \rangle_t(x, y)$ vs time-averaged adjusted retardance $\langle r' \rangle_t(x, y)$.

8 Analysis of Confocal Micrographs of Metaphase Plate

8.1 Chromosome Shape Parameters

To estimate the shapes of chromosome sections in the metaphase plate ($x = 0$ plane), we begin with the binarized sections shown in Fig. 4(a) of the Main Text. Let $\mathscr{C}_i$ be the geometrical region corresponding to chromosome section i; define $\mathbf{e_{i,1}}$ and $\mathbf{e_{i,2}}$ to be the eigenvectors of the moment of inertia tensor of $\mathscr{C}_i$ about its center of mass, with corresponding eigenvalues $e_{i,1}$ and $e_{i,2}$, where $e_{i,1} > e_{i,2}$. The orientation of the best fit ellipse is given by $\mathbf{e_{i,1}}$ while its major and minor axes are given by

$$a_i = 2\Big(\frac{e_{i,1}^3}{e_{i,2}}\Big)^{1/8}; \qquad b_i = \frac{2e_{i,1}}{(a/2)^3}.$$

The results of this procedure, applied to a set of binarized chromosomes sections, are shown in Fig. S10, which also gives histograms of chromosome shape parameters for $n = 11$ MII spindles. For a given spindle, the average shape parameters are defined as follows:

$$a \equiv \langle a_i \rangle_{i=1\ldots n_{\text{chr}}}; \qquad b \equiv \langle b_i \rangle_{i=1\ldots n_{\text{chr}}}, \tag{S10}$$

where n_{chr} is the number of identified chromosome sections in that spindle.

8.2 Definition of $g_{II}(s)$

To quantify spatial correlations in pixel intensity in binarized images, we use the intensity-intensity correlation function $g_{II}(s)$, an adaptation of the pair correlation function commonly used in condensed matter physics (*60*). Let $I(i, j)$ be the value of the pixel at position $\{i, j\}$ in an image; in a binarized image, $I(i, j) = 0$ or 1 (Fig. S11). To calculate $g_{II}(s)$, we consider all pixels $I(i', j')$ where $\{i', j'\}$ lies in an annulus, centered at $\{i, j\}$, of inner radius s and outer radius $s + ds$, where ds is of order one pixel. Let $\psi_s(i, j)$ be the fraction of white pixels in the annulus, $\psi_s(i, j) = \langle I(i', j') \rangle_{s<||\{i,j\}-\{i',j'\}||<s+ds}$. Then, $g_{II}(s)$ is the average of $\psi_s(i, j)$ over

all white pixels, i.e. all $\{i, j\}$ with $I(i, j) = 1$, normalized by the fraction of white pixels over the whole image.

9 Simulations of Chromosome Configurations

9.1 Generating Randomized Non-Overlapping Chromosome Configurations

To compare the observed chromosome configuration with the configuration we would expect if non-interpenetrating chromosomes were randomly positioned in the metaphase plate, we performed a series of Monte Carlo simulations to generate randomized chromosome configurations. The code takes as input a set of n_{chr} binarized chromosome sections, $\{\mathscr{C}_i\}_{i=1\ldots n_{\text{chr}}}$, where $\mathscr{C}_i$ is the geometrical region corresponding to chromosome section i, as well as a region $\mathscr{M}$ corresponding to the metaphase plate (Main Text Fig. 4, Fig. S12). In the *Mathematica* programming language, the `Region[]` function provides a convenient way to store and perform computations on the geometrical regions generated by image binarization. Let $\mathbf{c}_i$ and ϕ_i be the center of mass and orientation of the region $\mathscr{C}_i$. In pseudocode, our simulation proceeds:

Stage 1: Generate a Randomly Seeded Non-Overlapping Configuration of Chromosomes (Fig. S12(a-c))

1. Randomly place chromosome sections in the plane such that the center of mass of region $\mathscr{C}_i$ is $\mathbf{c}_i$ and its orientation is ϕ_i, where ϕ_i is defined relative to an arbitrary but fixed axis in $\mathscr{C}_i$. Centers and orientations are random variables defined such that

$$\mathbf{c}_i \text{ is uniformly distributed in } \mathscr{M}; \qquad \phi_i \text{ is uniformly distributed in } [0, 2\pi).$$

2. Define the total area of intersection of chromosome i with all other chromosomes and

with the spindle exterior,

$$A_{\text{int}}(i) = \sum_{j \neq i} A(\mathscr{C}_i \cap \mathscr{C}_j) + A(\mathscr{C}_i \cap \mathscr{M}^C),$$

where $\mathscr{M}^C$ is the complement of $\mathscr{M}$ and $A(\mathscr{R})$ is the area of region $\mathscr{R}$.

3. If $A_{\text{int}}(\mathscr{C}_1) = 0$, proceed to the next step. Otherwise, define another region, $\mathscr{C}_1'$, by rotating $\mathscr{C}_1$ by an angle $\Delta\phi$ about $\mathbf{c}_1$ and translating the resulting region by a vector $\Delta\mathbf{c}$, where $\Delta\phi$ and each component of $\Delta\mathbf{c}$ and are randomly drawn from Gaussian distributions with mean zero and amplitude $\Delta\phi_0$ and Δc_0 respectively. To minimize the run-time of the simulation, we set $\Delta c_0 = 2\,\mu$m and $\Delta\phi_0 = \pi/4$, but different choices for these parameters does not affect the statistics of the final configuration. If $A_{\text{int}}(\mathscr{C}_1') < A_{\text{int}}(\mathscr{C}_1)$, then set $\mathscr{C}_1 \to \mathscr{C}_1'$. Otherwise, proceed to the next step.

4. Repeat the previous step for chromosomes 2, ... n_{chr}. Then return to chromosome 1, and iterate until $A_{\text{int}}(\mathscr{C}_i) = 0$ for all i (Fig. S12(c))).

Stage 2: Monte Carlo Simulation: Generate Translational and Rotational Diffusion of Non-Overlapping Chromosomes (Fig. S12(c-d))

5. Let $\mathbf{c}_i^{\text{ini}}$ and ϕ_i^{ini} be the center of mass and orientation of region $\mathscr{C}_i$ at the completion of Stage 1.

6. Define a region, $\mathscr{C}_1'$, by rotating and translating $\mathscr{C}_1$ by $\Delta\phi$ and $\Delta\mathbf{c}$, as defined in Step 3. If $A_{\text{int}}(\mathscr{C}_1') = 0$, then set $\mathscr{C}_1 \to \mathscr{C}_1'$. Otherwise, proceed to the next step.

7. Repeat the previous step for chromosomes 2, ... n_{chr}.

8. Let $\mathbf{c}_i^{\text{new}}$ and ϕ_i^{new} be the center of the center of mass and orientation of chromosome $\mathscr{C}_i$. If the following three conditions are met: (i) For all i, $||\mathbf{c}_i^{\text{new}} - \mathbf{c}_i^{\text{ini}}|| > 0.1R_0$; (ii)

$\langle ||\mathbf{c}_i^{\text{new}} - \mathbf{c}_i^{\text{ini}}|| \rangle > 0.5R_0$; (iii) $\langle ||\phi_i^{\text{new}} - \phi_i^{\text{ini}}|| \rangle > \pi/4$, then set $\mathbf{c}_i^{\text{fin}} = \mathbf{c}_i^{\text{new}}$ and $\phi_i^{\text{fin}} = \phi_i^{\text{new}}$ and terminate the simulation. Otherwise, return to step 6.

Typical chromosome trajectories during Stage 2, plus the corresponding time course of the square displacement, are shown in Fig. S12(e & f).

9.2 Repulsive Ellipses: Simulation Framework for Arbitrary Potential $U(s)$

For director-aligned 3D voids with circular $x = 0$ cross-sections, we could model the pairwise interaction between them via a pairwise central potential $U(s)$, where s is the distance between void centers in the $x = 0$ plane. [1] In that case, for pairs of voids with centers located at yz-coordinates $\mathbf{s_i}$ and $\mathbf{s_j}$, the force from void i on void j would be given by

$$\mathbf{F}_{ij} = F(s_{ij})\hat{\mathbf{s}}_{ij} = -\frac{\partial U}{\partial s_{ij}}\hat{\mathbf{s}}_{ij},$$

where $s_{ij} \equiv |\mathbf{s}_j - \mathbf{s}_i|$ and $\hat{\mathbf{s}}_{ij} \equiv (\mathbf{s}_j - \mathbf{s}_i)/|\mathbf{s}_j - \mathbf{s}_i|$.

For voids with non-circular $x = 0$ cross-sections, long-range repulsive interactions generically produce torques as well as forces between voids, which predicts alignment between neighboring chromosome sections. This prediction is consistent with the co-alignment of neighboring chromosomes evident in micrographs of the metaphase plate (e.g. Main Text Figs. 3(b) & 4(a)). To model this effect, we represent the $x = 0$ void cross-sections as dimers, pairs of rigidly connected points separated by the length of the average chromosome semi-major axis $a/2$ (S.T. 8.1). Forces and torques arise from the interactions between the points comprising one dimer with points comprising another (Fig. S13(a)). More specifically, void section i is represented by two points $\mathbf{s}_i^1$ and $\mathbf{s}_i^2$, separated by the constant length $a/2$, i.e. $|\mathbf{s}_i^2 - \mathbf{s}_i^1| = a/2$. The center of mass of void section i is $\mathbf{s}^{\text{CoM}} = (\mathbf{s}_i^1 + \mathbf{s}_i^2)/2$ and its orientation ϕ_i is defined via

[1] Even for axisymmetric, tactoid-shaped voids, however, there is no guarantee that such a model would capture the physics of void self-organization: in general, forces mediated by 3D nematic elasticity are not pairwise additive.

the relation $\hat{\mathbf{s}}_i = 2(\mathbf{s}_i^2 - \mathbf{s}_i^1)/a \equiv \{\cos\phi_i, \sin\phi_i\}$. The force $\mathbf{F}_{ij}$ and torque τ_{ij} exerted on void section j by void section i are given by

$$\mathbf{F}_{ij} = F(|\mathbf{s}_j^{\text{CoM}} - \mathbf{s}_i^{\text{CoM}}|)\hat{\mathbf{s}}_{ij}; \qquad \tau_{ij} = \frac{1}{4}\sum_{m,n=1}^{2}((\mathbf{s}_j^n - \mathbf{s}_j^{\text{CoM}}) \times \mathbf{F}_{ij}^{mn}), \tag{S11}$$

where $\hat{\mathbf{s}}_{ij} = (\mathbf{s}_j^{\text{CoM}} - \mathbf{s}_i^{\text{CoM}})/|\mathbf{s}_j^{\text{CoM}} - \mathbf{s}_i^{\text{CoM}}|$ and $\mathbf{F}_{ij}^{mn}$ is the force from the point at $\mathbf{s}_i^m$ on the point at $\mathbf{s}_j^n$. Physically, this is equivalent to modeling a non-axisymmetric void as two axisymmetric voids that are rigidly attached to one another in the $x = 0$ plane.

To compare in detail the predictions of the above model with observed chromosome/void configurations in the metaphase plate, we performed simulations to find configurations of n_{chr} identical dimers in mechanical equilbrium, given a specific repulsive potential $U(s)$ and a randomized initial configuration of dimers within with an experimentally determined spindle boundary.[2] To do this, we used Eqn. S11 to numerically integrate the over-damped equations of motion for the dimer center of mass and orientation,

$$\dot{\mathbf{s}}_j^{\text{CoM}} = \frac{1}{\gamma}\left(\sum_{i\neq j}\mathbf{F}_{ij} + \mathbf{F}_{\text{bound},j}\right); \qquad \dot{\phi}_j = \frac{1}{\gamma_\phi}\sum_{i\neq j}\tau_{ij},$$

discretized as

$$\mathbf{s}_j^{\text{CoM}}(t+\Delta t) = \mathbf{s}_j^{\text{CoM}}(t) + \frac{\Delta t}{\gamma}\left(\sum_{i\neq j}\mathbf{F}_{ij}(t) + \mathbf{F}_{\text{bound},j}(t)\right); \qquad \phi_j(t+\Delta t) = \phi_j(t) + \frac{\Delta t}{\gamma_\phi}\sum_{i\neq j}\tau_{ij}(t), \tag{S12}$$

where $\mathbf{F}_{\text{bound},j}$ is a force exerted by the boundary on dimer j, and we chose the drag coefficients γ and γ_ϕ and time step Δt such that for later time-steps $|\Delta\mathbf{s}_j^{\text{CoM}}| \ll a/2$ and $\Delta\phi_j \ll \pi$. In early time-steps, randomly positioned particles can be arbitrarily close together and so can experience very large forces and torques; we therefore explicitly limit the maximum displacement of each component of a dimer's center of mass to Δx_{max} and its angular rotation to $\Delta\phi_{\text{max}}$; this

[2] A set of dimers may have many such equilibria, corresponding to different local minima of the total energy, and our simulations do not in general find the dimer configuration that globally minimizes the total energy of the system. However, all mechanical equilibria we find generate similar configuration statistics (Fig. S14).

only affects the first few time-steps of the evolution. In Eqns. S12, the term $\mathbf{F}_{\text{bound},j}$ is included to prevent repulsive dimers escaping the metaphase plate. We implement this confining force by placing N_{bound} equally-spaced points $\mathbf{b}_k$ along the experimentally determined boundary of the metaphase plate (Fig. S13(b)), each of which repels the dimer j with a force derived from the Yukawa potential $U^B(s) = B_0 e^{-s/\lambda_B}/s$,

$$\mathbf{F}_{\text{bound},j} = \sum_{k=1}^{N_{\text{bound}}} \mathbf{F}_{kj}^B; \qquad \mathbf{F}_{kj}^B = -\frac{\partial U^B(s_{kj})}{\partial s_{kj}} \hat{\mathbf{s}}_{kj},$$

where s_{kj} is the distance between boundary particle k and the center of mass of dimer j, $\hat{\mathbf{s}}_{kj}$ is the unit vector along the line joining these points, and the parameters B_0 and λ_B determine the amplitude and range of the Yukawa repulsion. We choose B_0 and λ_B to ensure that the average area of the convex hull of the final configuration of ellipses matches, within 10%, the average area of the convex hull of the experimentally observed chromosome sections.

We time-evolve the initial configurations using Eqn. S12 until the absolute values of the displacements of the particles comprising all dimers sum to a value less than a tolerance $tol \ll a/2$. The values of all parameters used in our simulations are given in Table S2. Finally, to convert the output of a simulation into a form that can be compared with experimental data, we replace the dimers with ellipses whose shape parameters a and b are derived from the specific experimental data set that was used in that simulation (Fig. S13(c)).

9.3 Results for Specific Long-Range Repulsive Potentials

The framework provided in S.T. 9.2 applies for arbitrary repulsive interactions in the $x = 0$ plane. To obtain the results presented in Main Text Fig. 4(e), we used the potential $U(s) = A_5 s^{-5}$, where the value of A_5 and other simulation parameters are given in Table S2. We also used the arbitrarily chosen potentials $A_3 s^{-3}$, $B_1 e^{-s/\lambda_B} s^{-1}$, and the potential $U_{\text{int}}(s)$ given in Eqn. S13 derived from the xy-plane model presented in S.T. 10.1, with the parameter s now

	Parameter	Description	Value	Unit
all sims	N_{bound}	number of boundary points	200	1
	$B_0\Delta t/\gamma$	effective amplitude of boundary repulsion	10^4	μm^3
	λ_B	range of boundary repulsion	0.1	μm
	tol	simulation stops when total motion is less than this value	10^{-5}	μm
	Δx_{max}	largest $\hat{\mathbf{y}}$ or $\hat{\mathbf{z}}$ displacement each dimer can make in a time-step	10^{-2}	μm
	$\Delta\phi_{\text{max}}$	largest angular displacement each dimer can make in a time-step	10^{-1}	rad
$U(s) = U_{\text{int}}(s)$ (Eqn. S13)	α	+1/2 spacing in defect quadrupole	9.9	μm
	β	-1/2 spacing in defect quadrupole	11.0	μm
	$8\pi k\Delta t/\gamma$	effective elastic constant	1.26	μm^2
$U(s) = A_3 s^{-3}$	$A_3\Delta t/\gamma$	effective amplitude of repulsion	10^{-1}	μm^5
$U(s) = A_5 s^{-5}$	$A_5\Delta t/\gamma$	effective amplitude of repulsion	0.5	μm^7
$U(s) = A_Y e^{-s/\lambda_Y} s^{-1}$	$A_Y\Delta t/\gamma$	effective amplitude of repulsion	0.02	μm^3
	λ_Y	range of Yukawa repulsion	1	μm

Table S2: Parameters for repulsive ellipse simulations. An entry of "1" in the Unit column indicates that a parameter is unitless. In all simulations, we set $\gamma_\phi = (\frac{a}{4})^2\gamma$.

interpreted as the distance between two points in the yz-plane. All simulation parameters are given in Table S2.

9.4 Comparison of Experimental and Simulated Configurations: Positional Correlations

It is immediately visually evident that simulated random chromosome configurations are very different to experimentally observed configurations, while all four repulsive ellipsoid simulations produce configurations that share several features with experimental ones, including well-separated chromosomes/ellipses, and the formation of a ring of $\sim$15 mostly radially oriented chromosomes ellipses near the spindle boundary (Fig. S14(a)). To quantitively compare these configurations, we measured three parameters: the positions of the trough and peak in $g_{II}(s)$ and the minimum distance $w_{\text{min}}(i)$ between the surface of chromosome section i and the

surface of its nearest neighbor (Fig. S14(a, *Inset*)). In terms of these parameters, randomized configurations are very different from experimentally observed ones (Main Text Fig. 4(c) & Fig. S14(b-d)), while configurations derived from the repulsive ellipsoid simulations are similar (Fig. S14(d)). We note, however, that in the simulated repulsive data, peaks and troughs in $g_{II}(s)$ have a larger amplitude than in the experimental $g_{II}(s)$, while the modal value of w_{min} is $\sim$10% higher. These discrepancies presumably occur because the dimers in each simulation are of identical size and shape, whereas chromosomes/voids in a real spindle is not; this may facilitate more efficient ordering of simulated dimers compared to metaphase chromosomes (*61*).

9.5 Comparison of Experimental and Simulated Configurations: Orientation of Outer Chromosomes Relative to Spindle Boundary

We also quantified the tendency of the outermost ring of chromosomes to orient with their long axes normal to the the spindle boundary (Fig. S15). This trend is seen in all 11 metaphase confocal micrographs, as well as in the simulated data. In the simulation, this phenomenon arises because of a combination of factors: ellipses exert a torque on one another that tends to align them, while the confining boundary exerts no torque by construction (S.T. 9.2). When many ellipses are confined in this way, this leads to the formation of an outer ring of radially oriented ellipses. However, the torque-free boundary condition in our simulations is not derived from known spindle physics; rather, we use it because it replicates the data well. The same combination of forces and torques may explain the same trend in the observed chromosome configurations, but, in the absence of a detailed model for how chromosomes interact with the spindle boundary, we are unable to directly test this hypothesis.

10 Analytical Model for Repulsion Between Voids in 2D Nematic ($z = 0$ Plane Model)

10.1 Expression for Deformation Field Around Parallel Voids & Associated Interaction Energy

In a 2D nematic with equal elastic constants, the general formula for the orientation of the director in the vicinity of a collection of topological defects of strength σ_i positioned at $z_i \equiv x_i + iy_i$ is given by the formula (*62, 63*):

$$\theta(x, y) = \frac{1}{2i}(\log \Omega(z) - \log \Omega(\bar{z})),$$

where $\Omega(z) = \prod_i (z - z_i)^{\sigma_i} + \psi_0$, and the constant ψ_0 controls the director orientation at infinity. Under the assumption that the defects are separated by distances much larger than the core size a_0 , the energy stored in the field is given by

$$U = -8\pi k \sum_{i \neq j} \sigma_i \sigma_j \log \frac{z - z_i}{a_0} + C,$$

where the constant C includes the core energies of all defects (*63*).

We describe the deformation field due to an isolated 2D void as that generated by a single row of four defects, comprising two $-1/2$ defects separated by a distance β and two $+1/2$ defects separated by a distance α, with $\alpha < \beta$. All defects lie along the line $y = 0$ (Main Text Fig. 4(d, *left*) & Fig. S16). Setting $\psi_0 = 0$ realizes our desired far-field value $\theta = 0$. The explicit expression for the director orientation (thin gray curves in figures) is

$$\theta(x, y) = \frac{1}{2}\Bigg(- \arg\left(1 + \frac{4iy}{-2x - 2y + \alpha}\right) - \arg\left(1 + \frac{4iy}{2x + 2y + \alpha}\right) + \arg\left(1 + \frac{4iy}{-2x - 2y + \beta}\right) + \arg\left(1 + \frac{4iy}{2x + 2y + \alpha}\right)\Bigg)$$

and the corresponding energy is given by

$$U_1 = -4\pi k \log \frac{16 a_0^2 \alpha \beta}{(\alpha^2 - \beta^2)^2} + C.$$

For two parallel rows of defects, both centered at $x = 0$ and separated by a vertical distance d (Main Text Fig. 4(d, *left*)), the total energy is given by

$$U_2(d) = -8\pi k\left(2\log\left(\frac{d}{a_0}\right)+\log\left(\frac{256a_0^4\alpha\beta\sqrt{(\alpha^2+d^2)(\beta^2+d^2)}}{(\alpha-\beta)^2(\alpha+\beta)^2(\alpha^4-2\alpha^2(\beta^2-4d^2)+(\beta^2+4d^2)^2)}\right)\right)+2\,C,$$

and the energy due to interactions between the voids is given by

$$U_{\text{int}}(d) = U_2(d) - 2U_1 = -8\pi k\log\left(\frac{16d^2\sqrt{(\alpha^2+d^2)(\beta^2+d^2)}}{(\alpha^4-2\alpha^2(\beta^2-4d^2)+(\beta^2+4d^2)^2)}\right). \quad \text{(S13)}$$

In the far field ($d \gg \alpha$, $d \gg \beta$), this interaction takes the simple form

$$U_{\text{int}}(d) \approx \frac{3\pi\alpha^2\beta^2}{d^4}. \quad \text{(S14)}$$

10.2 Interaction Between Voids is Always Repulsive

Evaluating the derivative $\mathrm{d}U_{\text{int}}/\mathrm{d}d$ and completing all available squares shows that $\mathrm{d}U_{\text{int}}/\mathrm{d}d < 0$ for all positive values of $\alpha, \beta,$ and d.

10.3 Relationship Between Topological Defect Configuration and Void Boundary Shape

Given specific values of α and β, we define voids in our model as the 2D regions enclosed by those integral curves of the vector field $\{\cos\theta(x,y), \sin\theta(x,y)\}$ that pass through both -1/2 defects (Main Text Fig. 4(d, *top left*), thick black curve). This construction yields tactoid-like void shapes while ensuring that tangential boundary conditions are satisfied on the void surfaces. However, since the integral curves are not exact circle arcs, we must choose the void spacing parameters α and β to match the experimentally observed tactoid shape (Main Text Fig. 3(f)). To determine the values of the parameters of $U_{\text{int}}(s)$ given in Table S2, we set β equal to the experimentally measured void length ($\beta = 11.0\,\mu$m), and choose the parameter α such that the curvature of the deformation field at the void boundary matches the radius of curvature of the experimentally observed voids ($\alpha = 9.9\,\mu$m; Fig. S16).

Supplemental Figures S1-S16

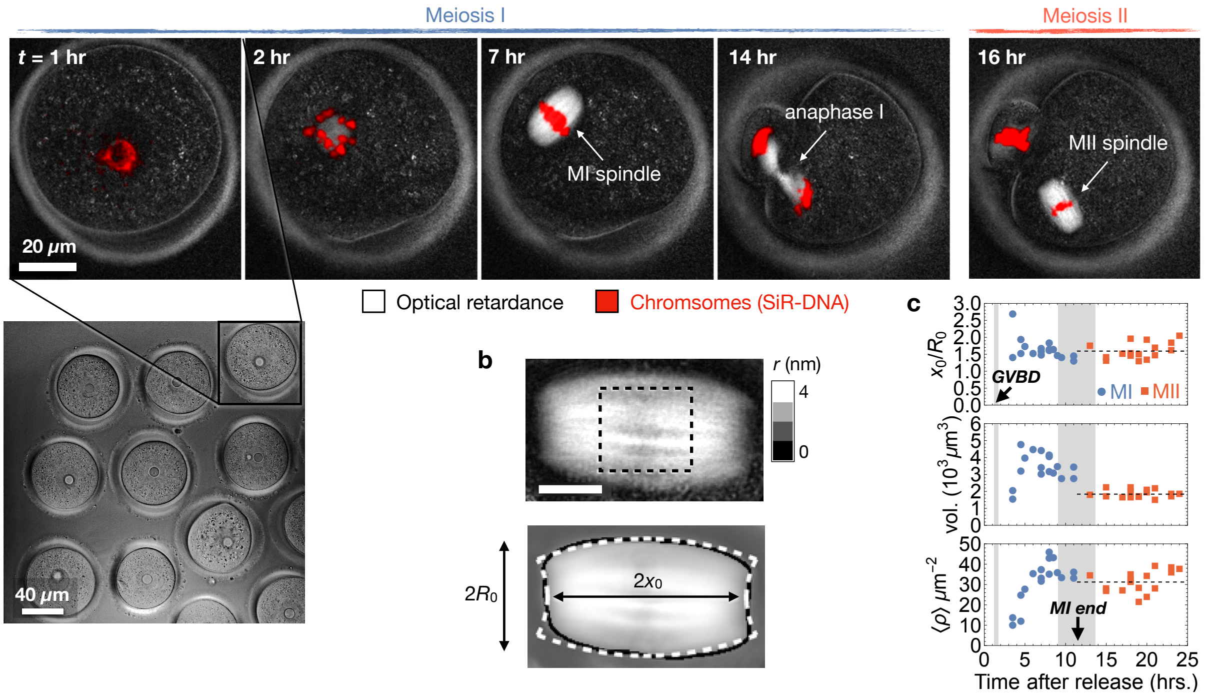

Figure S1: **With respect to the parameters measured by the LC-PolScope, MII oocytes remain in steady state for at least 12 hours after completion of meiosis I.** (a) *Main Panel:* GV-arrested oocytes. *Inset:* Time series of maturation of an individual oocyte, showing the formation of the first and second (MI and MII) meiotic spindles, as well as anaphase I. Times are given relative to release from meiotic arrest (Materials & Methods). Spindles are imaged using LC-PolScope (grayscale); chromosomes are stained with SiR-DNA and imaged using epifluorescence (red). (b) For both MI and MII spindles, length $2x_0$ and maximum width $2R_0$ are measured from the best-fit pole-indented tactoid (Materials & Methods). (c) Plots showing the time-course of the spindle's aspect ratio x_0/R_0, volume, and microtubule cross-section density $\langle \rho \rangle$ (Eqn. S1). Release of spindles from meiotic arrest occurs at $t = 0$. Grey bars indicate the distributions of times required to complete GV breakdown and times required to complete meiosis I (mean $\pm$ standard deviation; $n = 71$ oocytes). Density is calculated using the retardance measured in an $8\,\mu$m $\times$ $8\,\mu$m box at the center of each spindle (dashed black square in (b)), and sample thickness equal to the spindle diameter, $T = 2R_0$. For MI spindles (blue circles), density and volume grow from zero over the course of several hours. For MII spindles (orange squares), all parameters reach steady-state $\lesssim$ 1 hr after completion of meiosis I, and no trend is evident for at least 12 hrs thereafter, and are consistent with Gaussian distribution about the mean (black dashed line, Kolmogorov Smirnov test with $p > 0.05$).

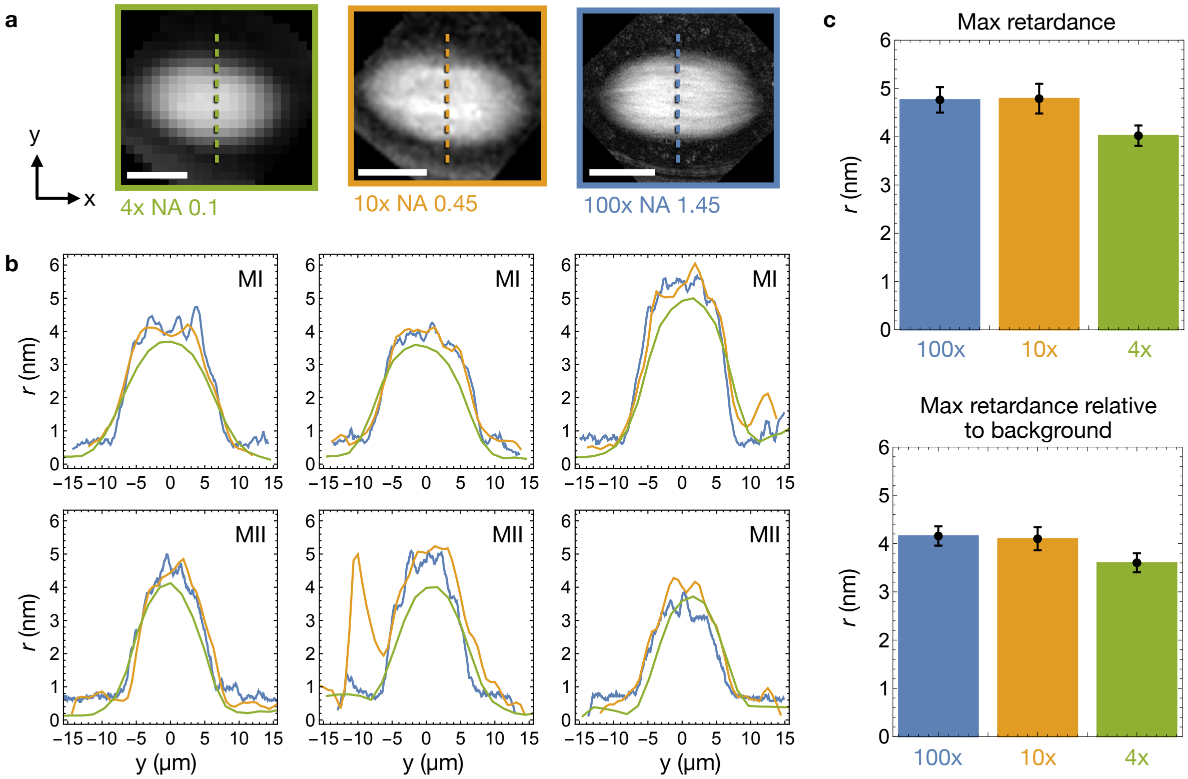

Figure S2: **LC-PolScope retardance profile of spindles does not depend on NA of optical system, consistent with collimated light assumption.** (a) The same MI spindle imaged with three different objective lenses: 4x (NA 0.3); 10 x (NA 0.7); 100x (NA 1.4). Scale bars 10 μm. (b) Retardance profiles through the center of three MI spindles (*top row*) and three MII spindles (*bottom row*), in the direction perpendicular to the spindle axis (dashed lines in (a)). (c) Histograms of the maximum retardance in the three MI line profiles shown in (b). The maximum measured retardance is similar in all cases, as is the maximum retardance with the background retardance values subtracted. Error bars indicate SE.

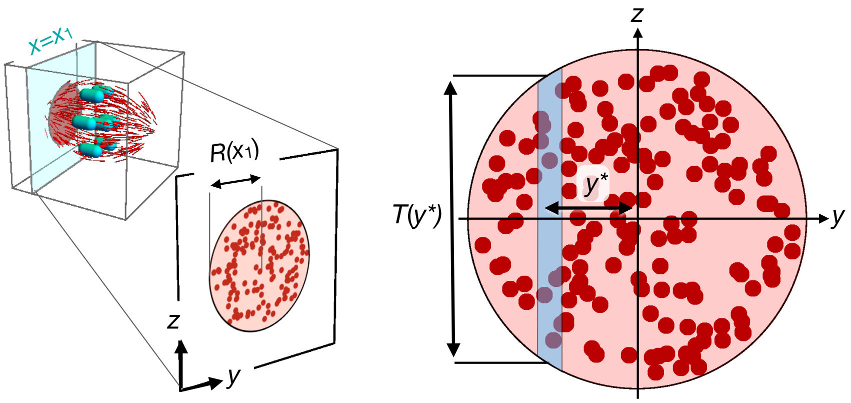

Figure S3: **Thickness profile of spindle with long axis $\hat{x}$ perpendicular to optical axis $\hat{z}$.** In the plane $x = x_1$, the spindle cross-section has radius $R(x_1)$, and the spindle boundary is described by the equation $y^2 + z^2 = R(x_1)^2$. The optical path length T through the spindle along the ray $y = y^*$ is given by $T(y^*) = 2(R(x_1)^2 - (y^*)^2)^{1/2}$.

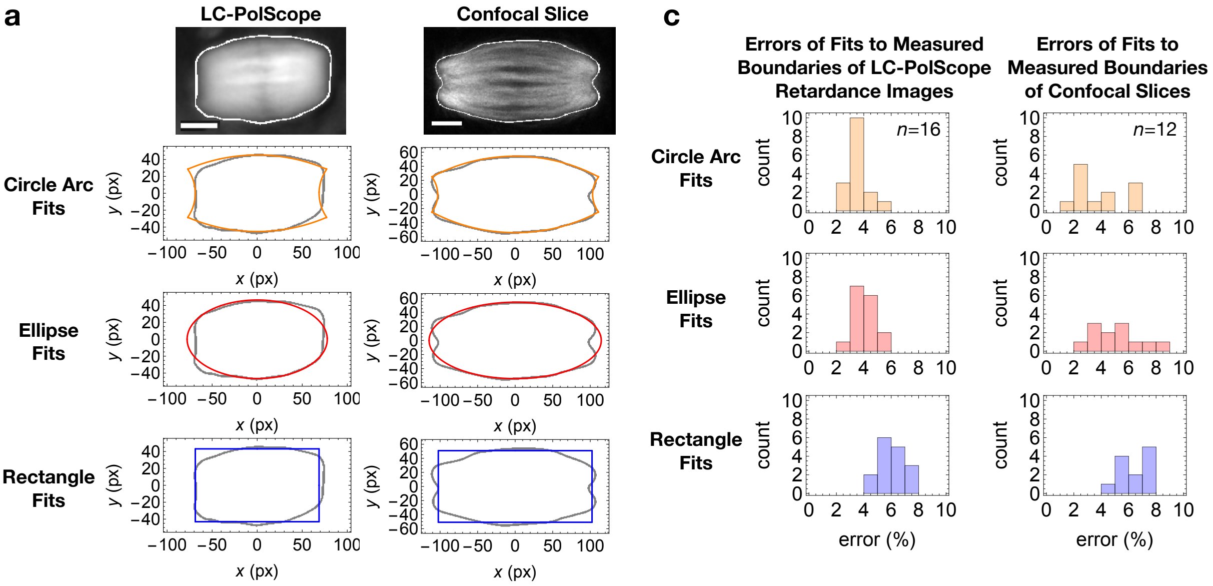

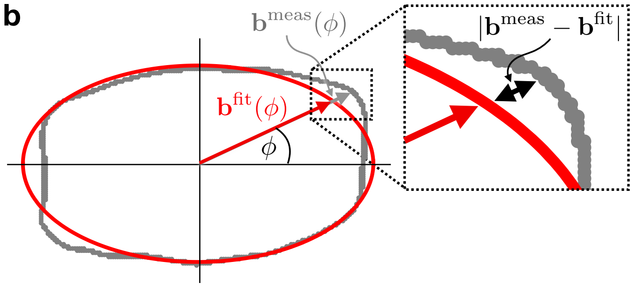

**d**	**LC-PolScope (*n*=16)**	**Confocal (*n*=12)**
**% Error of Circle Arc Fits**	3.5 ± 0.2	3.8 ± 0.4
**% Error of Ellipse Fits**	4.0 ± 0.2	5.1 ± 0.4
**% Error of Rectangle Fits**	6.1 ± 0.2	6.4 ± 0.2

Figure S4: **Circle arc fits are a better approximation to MII spindle boundaries than ellipse or rectangle fits.** (a) Three different fits to the empirically measured spindle boundaries of LC-PolScope retardance images (*left column*) and confocal slices containing the spindle long axis (*right column*). *Top row:* Spindle boundaries shown as thin white curves (Materials & Methods). Scale bars 5 μm. *Bottom three rows:* Fits of the measured boundaries to circle arc segments (Fig. S5), ellipses, and rectangles. (b) After expressing a given fit $\mathbf{b}^{\text{fit}}(\phi)$ and the measured boundary $\mathbf{b}^{\text{meas}}(\phi)$ in polar coordinates in the xy-plane, the fractional error associated with the fit, at a specific polar angle ϕ, is given by $|\mathbf{b}^{\text{fit}}(\phi) - \mathbf{b}^{\text{meas}}(\phi)|/|\mathbf{b}^{\text{meas}}(\phi)|$, and the average error over all angles is $\frac{1}{2\pi}\int_0^{2\pi} |\mathbf{b}^{\text{fit}}(\phi) - \mathbf{b}^{\text{meas}}(\phi)||\mathbf{b}^{\text{meas}}(\phi)|^{-1}\mathrm{d}\phi$. (c) Histograms of average errors of each type of fit for all spindles we imaged, expressed as percentages. (d) Means and standard errors of the histograms shown in (c). For both LC-PolScope and confocal data, circle arcs provide better fits than ellipses or rectangles.

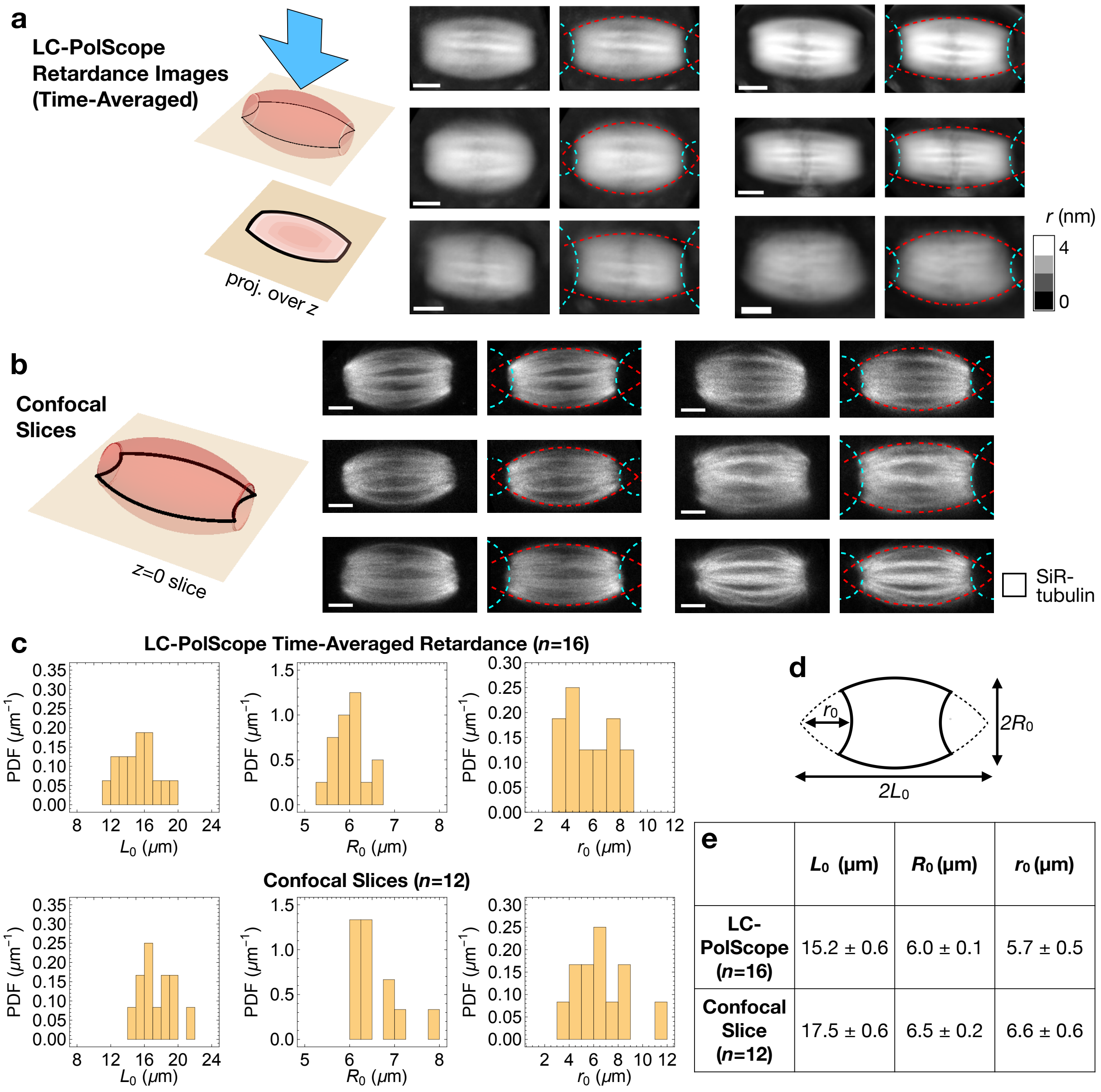

e	L_0 (μm)	R_0 (μm)	r_0 (μm)
LC-PolScope (*n*=16)	15.2 ± 0.6	6.0 ± 0.1	5.7 ± 0.5
Confocal Slice (*n*=12)	17.5 ± 0.6	6.5 ± 0.2	6.6 ± 0.6

Figure S5: **Spindle shape parameters from confocal slices vs spindle shape parameters from LC-PolScope.** (a) ***Main Panel:*** LC-PolScope time-averaged retardance images $\langle r \rangle_t$ (*left panels of image pairs*), which we interpret as $\hat{\mathbf{z}}$-projections of microtubule cross-sectional density, and the corresponding best fit circle arc segments (*right panel*, dashed colored lines). ***Cartoon Inset:*** For spindles with concave polar caps, the $\hat{\mathbf{z}}$-projection implicit in LC-Polscope image acquisition obscures the true shape of the spindle cross-section. (b) ***Main Panel:*** Slices of confocal stacks that contain the spindle's central axis (*left panels*), and the corresponding best fit circle arc segments (*right panels*, dashed colored lines). Many, but not all, of the spindles we imaged have visually evident concave polar indentations. ***Cartoon Inset:*** Confocal slices containing the spindle long axis allow more accurate measurement of the spindle cross-section. (c) Histograms of best-fit shape parameters for $n = 16$ LC-PolScope images and $n = 12$ confocal slices. (d) The generatrix of a pole-indented tactoid is made up of the four circle arc segments shown, which are fully determined by the three shape paramaters L_0, R_0, and r_0. (e) Shape parameters found by fitting LC-PolScope retardance images are systematically $\sim 10\%$ smaller than those found by fitting confocal slices ($p < 0.01$ for L_0 and R_0; two-tailed Student's t-test). All scale bars 5 μm.

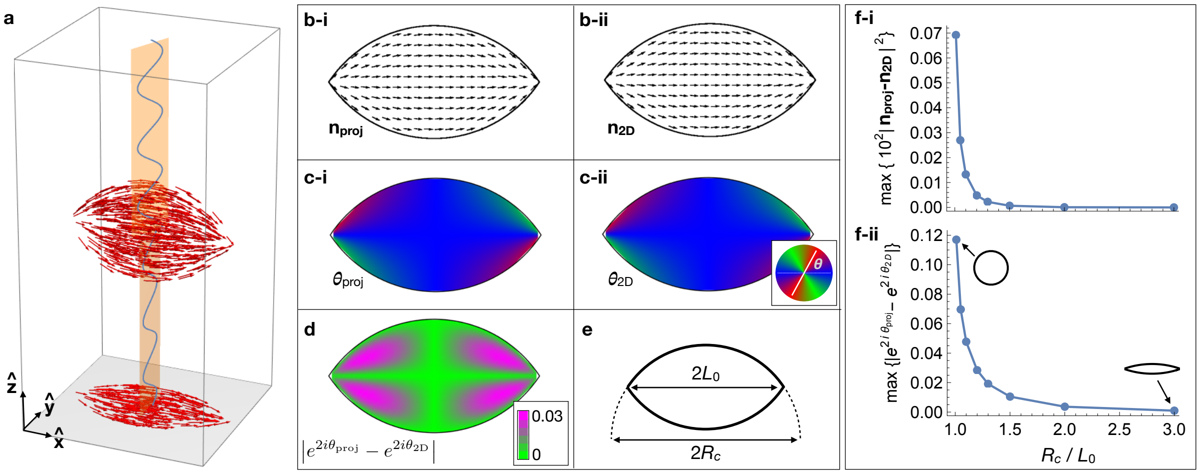

Figure S6: **Comparison of 3D nematic fields projected into 2D, and 2D nematic fields obtained from a 2D model.** (a) At each pixel of a 2D image, the LC-PolScope measures the nematic field averaged over the thickness of the spindle; (b) The nematic field obtained by (i) assuming a 3D circle arcs model, integrating over the optical axis $\hat{\mathbf{z}}$, and normalizing the resulting field, and (ii) a 2D version of the circle arcs model. (c) Angles obtained from the fields in (b). (d) Absolute difference in 2D nematic order parameter between the fields in (c). (e) All data in this panel is generated from a model in which spindle geometry fully is characterized by the two parameters R_c and L_0. In terms of the parameters of the pole-indented tactoid (Fig. S5), $R_c = (L_0^2 + R_0^2)/2R_0$. (f) The maximum absolute difference between the fields in (b) and the fields in (c) and is greatest for the smallest values of the spindle aspect ratio R_c/L_0. In oocyte spindles, $R_c/L_0 \approx 1.5$ (Fig. S5), and the maximum error between the 2D and 3D-projected nematic fields is about 1%.

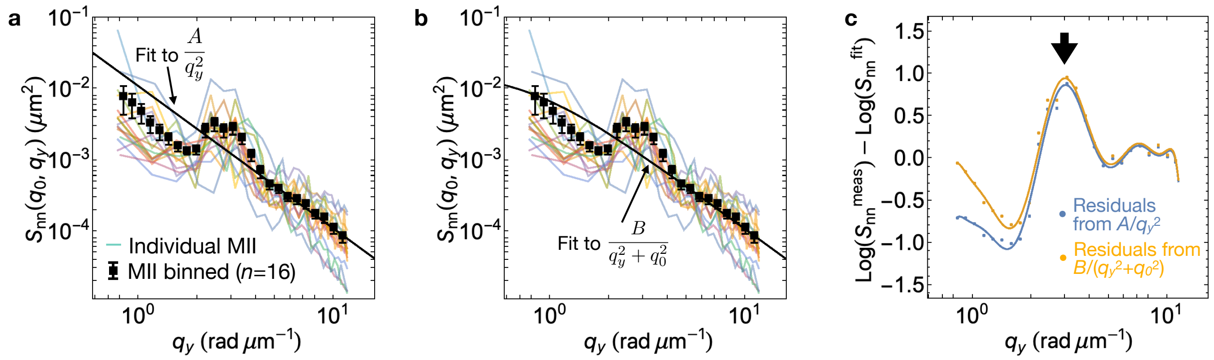

Figure S7: **Fits of theoretical predictions of s_{nn} to s_{nn} calculated from LC-PolScope fluctuation data.** (a) Fit of avergage experimentally determined $s_{\mathrm{nn}}(q_0, q_y)$ (black points with error bars) to theoretical prediction, assuming $q_0 \to 0$ (Eqn. S8). (b) Fit of avergage experimentally determined $S_{\mathrm{nn}}(q_0, q_y)$ to theorietical prediction, assuming $q_0 = 2\pi/\lambda_0$ (Eqn. S7), where $\lambda_0 = 8\,\mu$m is the size of the analysis box. (See Main Text Fig. 2.) (c) The location of the peak (black arrow at $q_y = 3.0$ rad μm^{-1}) is identified by fitting the residuals (blue and yellow points) of the fits in (a) and (b), calculated in log space. Continuous curves are ad-hoc polynomial fits; the peak near $q_y = 3.0$ rad μm^{-1} is identified using Newton's method.

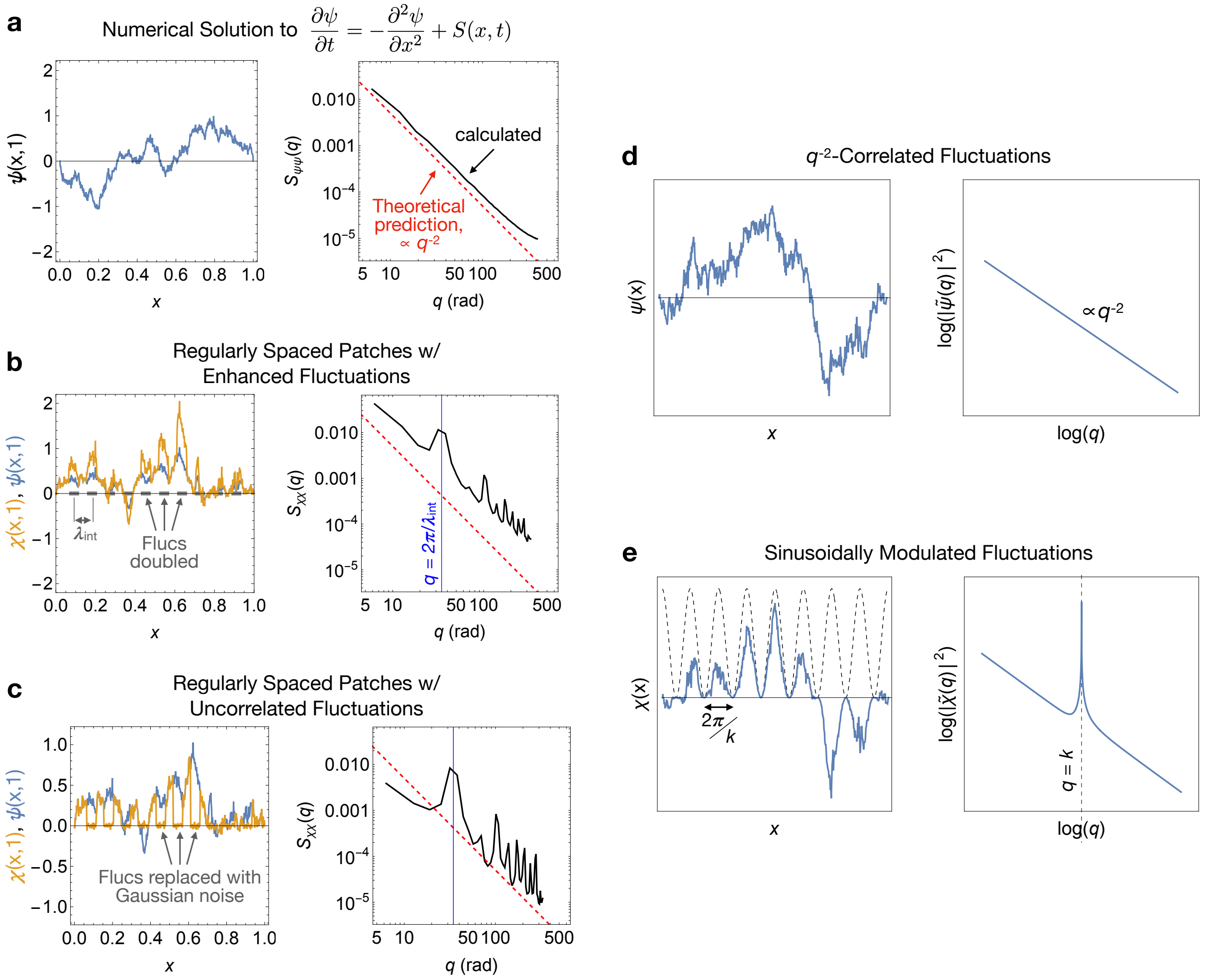

Figure S8: **Regularly spaced patches in a fluctuating background cause a peak in the equal-time correlation function at the wavenumber corresponding to the spacing between voids.** (a) *Left:* Numerical solution $\psi(x,t)$ of the (1+1)-D diffusion equation with non-conservative noise, plotted at a time $t = 1$ that is long enough for the simulation to sample the steady-state distribution of field configurations. *Right:* The calculated equal-time correlation function $S_{\psi\psi}(q)$ (black curve) closely matches the theoretical prediction, $S_{\psi\psi}(q) = 1/(2q^2)$ (red dashed line). (b) *Left:* Starting with the numerical solution $\psi(x,t)$, we define another field $\chi(x,t)$ that has larger fluctuations in $N = 10$ regularly spaced patches, each of width $w_0 = 0.05$ (grey bars along x-axis). *Right:* The corresponding equal-time correlation function has a peak at the q-value corresponding to the period λ_{int} of the patches. The overall amplitude $S_{\chi\chi}(q)$ of the curve is larger due to the larger fluctuations, but retains its q^{-2} shape at high and low q. The additional spikes at higher q are due to "ringing", a numerical artifact in which the sharp periodic jumps in $\chi(x,t)$ cause peaks at integer multiples of $2\pi/\lambda_{\text{int}}$ when the Fourier transform is calculated. (c) *Left:* Starting with the numerical solution $\psi(x,t)$, we define another field $\chi(x,t)$ that has Gaussian (i.e. uncorrelated) fluctuations in 10 regularly spaced patches. *Right:* The corresponding equal-time correlation function has a peak at the q-value corresponding to the period of the patches. (d) A field $\psi(x)$ has the power spectrum $|\tilde{\psi}(q)|^2 \propto q^{-1}$. (e) Modulating $\psi(x)$ by a sinusoidal function of period $2\pi/k$ leads to a peak in the power spectrum of the modulated function at $q = k$, and q^{-2} behavior at higher and lower q.

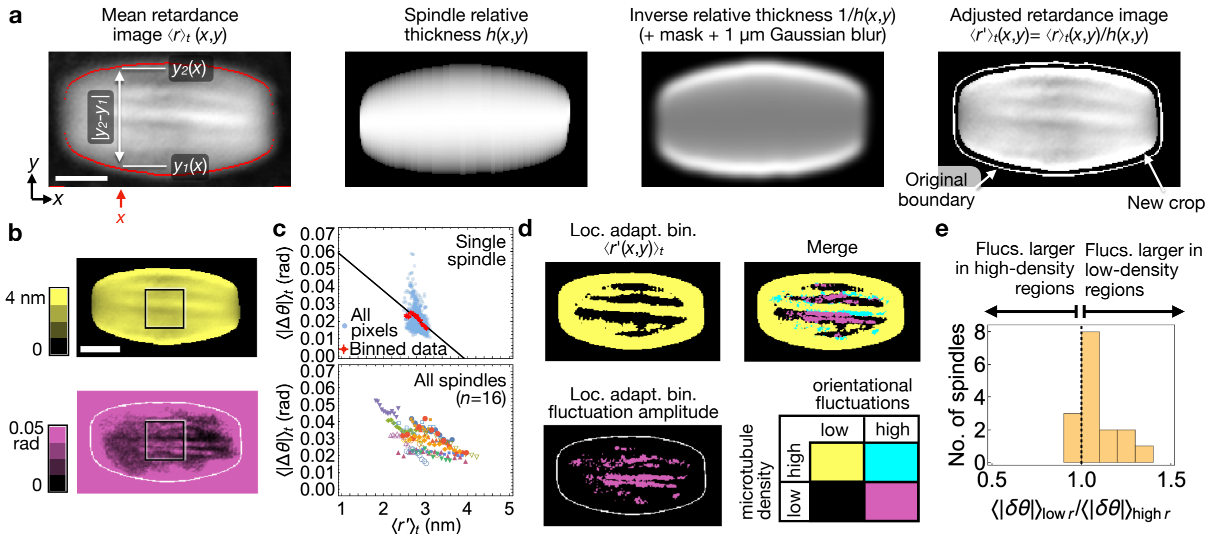

Figure S9: **Correlations between fluctuation magnitude and adjusted retardance.** (a) Finding the time-averaged retardance image $\langle r' \rangle_t(x, y)$ adjusted to take account of varying spindle thickness. From left to right, we first identify the spindle midpoint $(y_1 + y_2)/2$ and radius $|y_2 - y_1|/2$ at each point x along the spindle axis. From these values, we construct the normalized relative thickness image $h(x, y)$, which takes the value 1 at the midpoint between the vertical positions of the spindle boundary. We next find the inverse relative thickness image $1/h(x, y)$; to avoid pixelization artifacts, we first multiply the raw $1/h(x, y)$ image by the retardance mask (the region inside the boundary shown in the $\langle r \rangle_t(x, y)$ image), and convolve the resulting image with a Gaussian filter of radius $1\,\mu$m. We find the adjusted retardance image $\langle r' \rangle_t(x, y)$ by pixelwise multiplication and finally multiply this image by an updated retardance mask constructed by removing $1\,\mu$m from all boundaries of the original mask. (b-e) Once we have constructed $\langle r' \rangle_t(x, y)$, we repeat without further modification the analysis shown in Main Text Fig. 2.

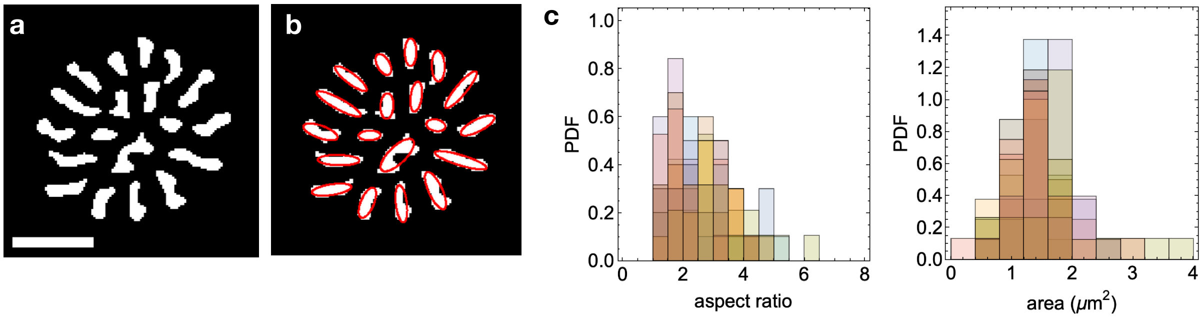

Figure S10: **Properties of chromosome sections in the metaphase plate.** (a & b) Best fit ellipses overlaid on binarized chromosome sections for one spindle. Scale bar $5\,\mu$m. (c) Distribution of chromosome aspect ratios a/b and areas in $n = 11$ spindles, in each of which we have identified $n_{\rm chr}$ =19 or 20 chromosome sections. The mean value of a/b is 2.5 ± 0.1, and the average chromosome area is $(1.45 \pm 0.03)\,\mu$m^2 (mean $\pm$ SE).

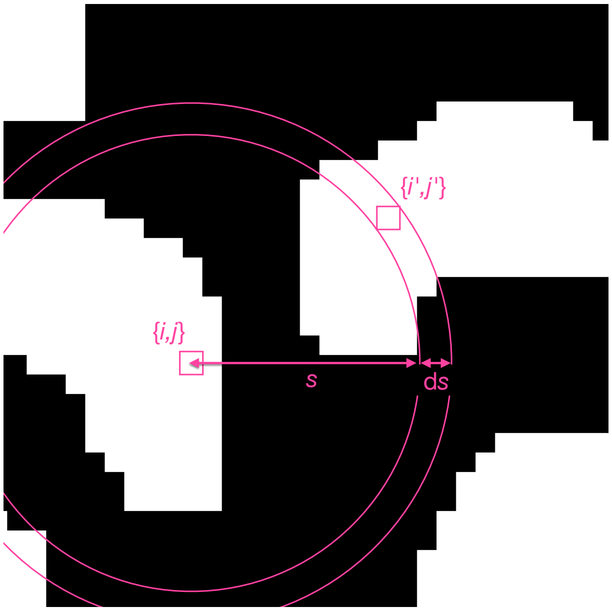

Figure S11: **Calculation of $g_{II}(s)$.** We first draw an annulus centered at $\{i,j\}$, with inner radius s and outer radius $s+ds$, and then calculate $\psi_s(i,j)$, the fraction of white pixels in the annulus. Then, $g_{II}(s)$ is the average of $\psi_s(i,j)$ over all white pixels, i.e. all $\{i,j\}$ with $I(i,j)=1$, normalized by the fraction of white pixels over the whole image.

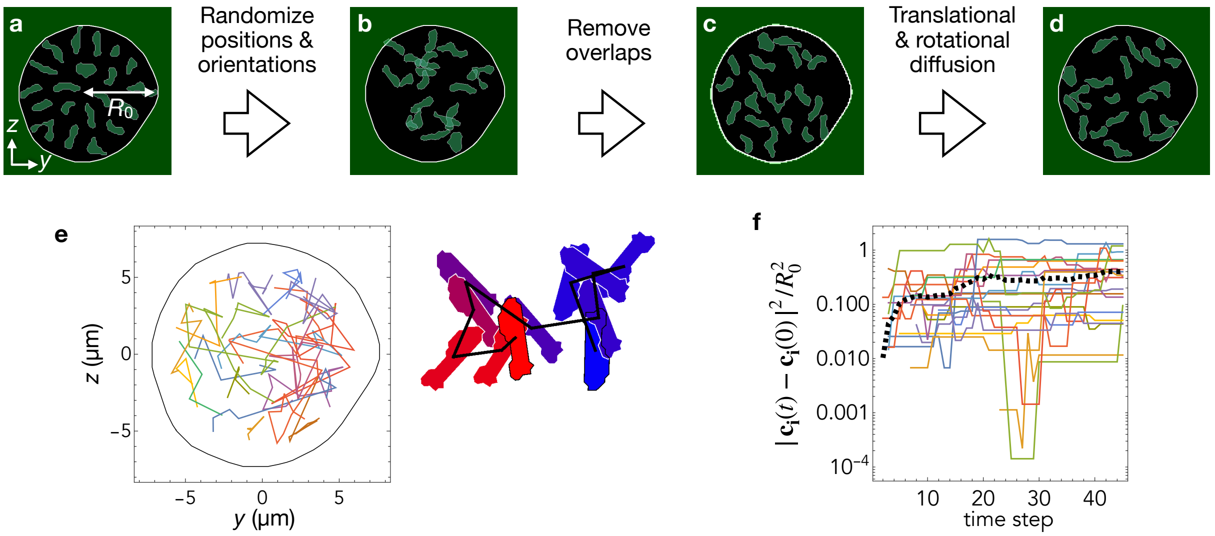

Figure S12: **Overview of simulations to generate randomized non-overlapping chromosome configurations.** (a-b) We identify the outlines of n_{chr} chromosome sections and randomly place them inside the spindle boundary; (b-c) we translate and rotate chromosomes sections to reduce and ultimately eliminate the overlap of chromosome sections with each other and with the region outside the spindle boundary; (c-d) we randomly translate and rotate non-overlapping chromosome sections to generate free diffusion. (e) *Left:* Trajectories of all centroids $\mathbf{c_i}(t)$ during the free diffusion stage of the simulation. Each color corresponds to the trajectory of the centroid of one chromosome section. *Right:* Trajectory of a single chromosome section $\mathscr{C}_i$ during the free diffusion stage. Blue indicates the initial configuration; red indicates the final configuration; the black line indicates the trajectory of the centroid $\mathbf{c_i}(t)$. (f) Square displacement of the centroids, in units of the metaphase plate radius, defined here as $R_0 \equiv \sqrt{A_{MP}/\pi}$, where A_{MP} is the area inside the spindle boundary. The free diffusion stage of the simulation proceeds until the centroids of all sections have translated at least $0.1R_0$ and the average absolute displacement is at least $0.5R_0$. (Black dashed line in plot shows the average square displacement from initial position.)

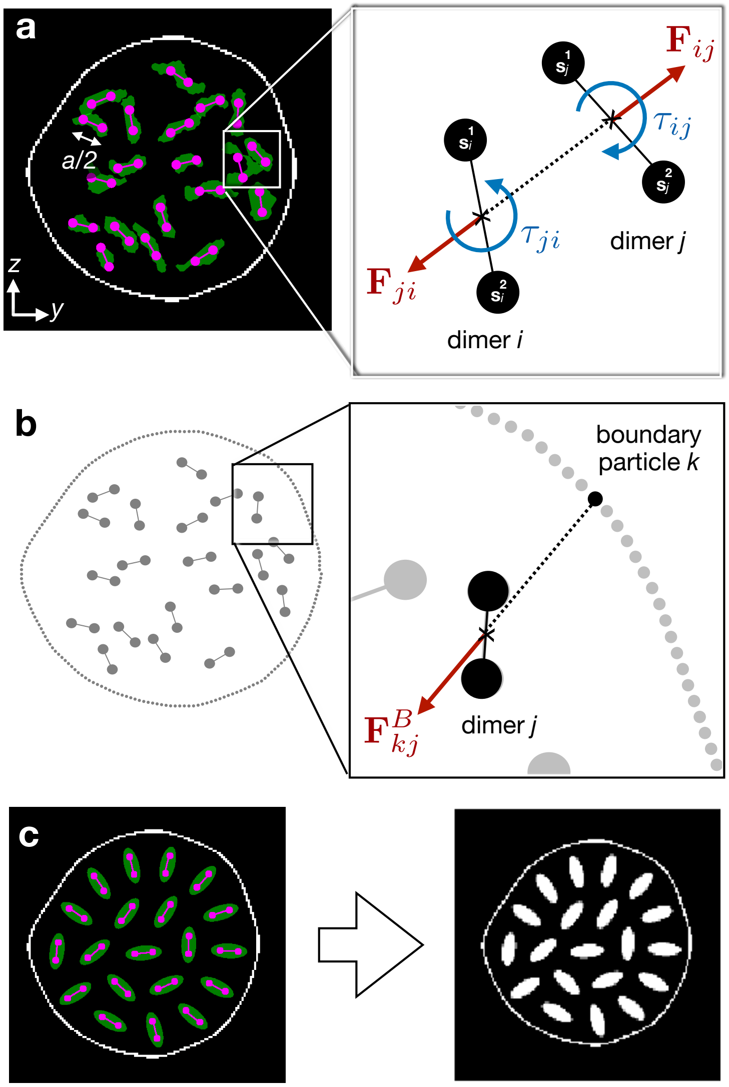

Figure S13: **Framework for repulsive ellipsoid simulations.** (a) Chromosome section j is represented as a dimer, a pair of points $\mathbf{s}_j^1$ and $\mathbf{s}_j^2$ separated by a fixed distance $a/2$, where a is the average chromosome long axis in the $x = 0$ plane. Forces and torques on dimer j from dimer i are inherited from the forces between the points comprising the dimers. (b) The boundary of the spindle is represented by a chain of N_{bound} equally-spaced points; each boundary point k exerts a force $\mathbf{F}^B_{kj}$ on the centroid of dimer j; the total force $\mathbf{F}_{\text{bound},j}$ from the boundary on dimer j is given by $\sum_{k=1}^{N_{\text{bound}}} F^B_{kj}$. (c) To compare simulated configurations with experimental ones at the end of the simulation, dimers are converted into identical ellipses with major and minor axes a and b.

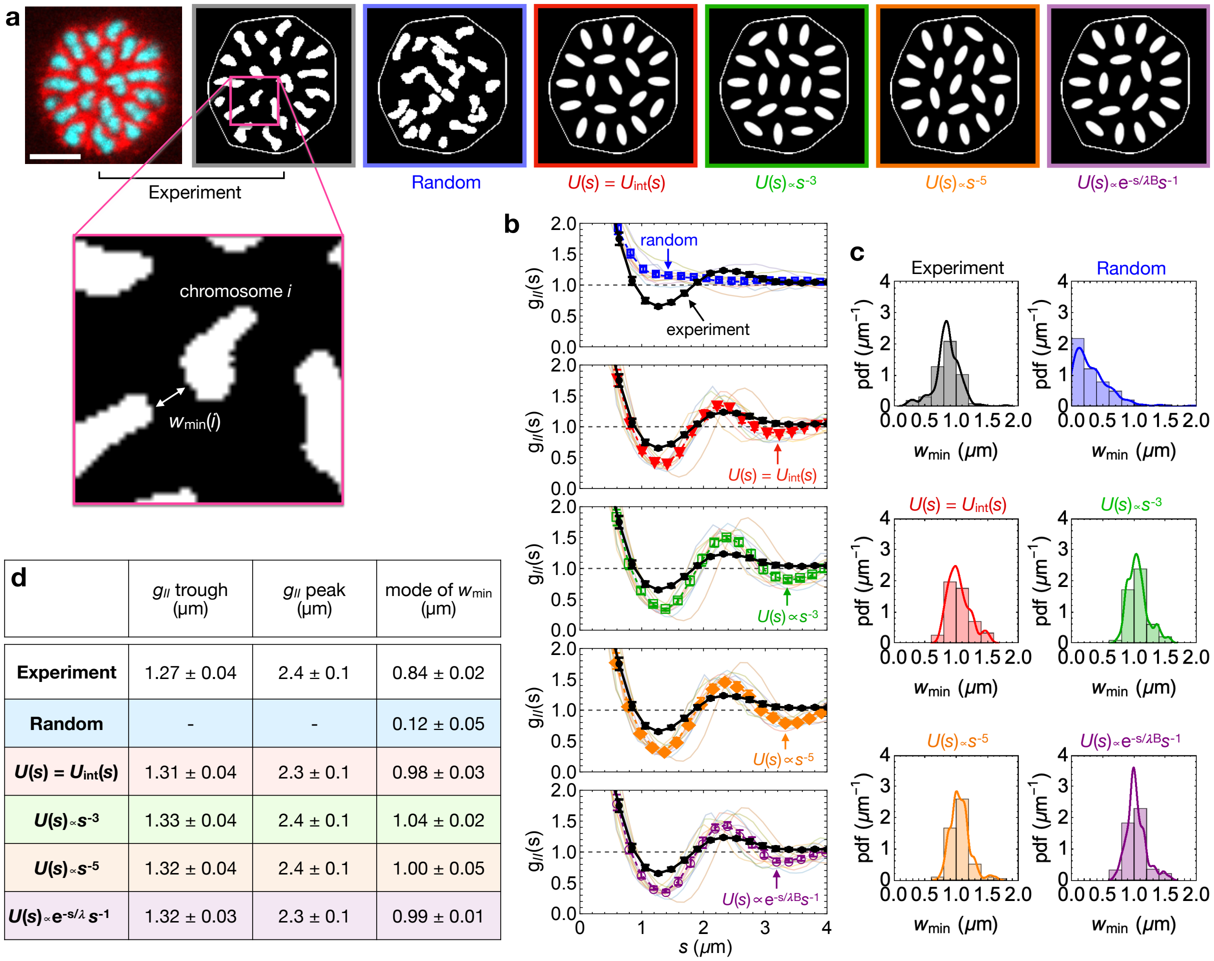

d	g_{II} trough (μm)	g_{II} peak (μm)	mode of w_{min} (μm)
**Experiment**	1.27 ± 0.04	2.4 ± 0.1	0.84 ± 0.02
**Random**	-	-	0.12 ± 0.05
**$U(s) = U_{int}(s)$**	1.31 ± 0.04	2.3 ± 0.1	0.98 ± 0.03
**$U(s) \propto s^{-3}$**	1.33 ± 0.04	2.4 ± 0.1	1.04 ± 0.02
**$U(s) \propto s^{-5}$**	1.32 ± 0.04	2.4 ± 0.1	1.00 ± 0.05
**$U(s) \propto e^{-s/\lambda} s^{-1}$**	1.32 ± 0.03	2.3 ± 0.1	0.99 ± 0.01

Figure S14: **Comparison of simulated chromosome configurations with experimentally observed ones.** (a) From left to right: original and binarized experimental images of chromosomes in the metaphase plate (scale bar 5 μm); final configuration of a simulation where we randomized the positions of the binarized experimental chromosome sections; and the final configurations of four simulations of ellipses interacting via long-range repulsive potentials. All simulations are performed using the experimentally measured spindle boundary (white curve). In the long-range repulsion simulations, the major and minor axes of the ellipses shown are determined by the average experimentally measured chromosome shapes. *Inset:* For chromosome i, the nearest neighbor separation $w_{\rm min}(i)$ is the smallest distance between the surface of chromosome i and the surface of any other chromosome. (b) Intensity-intensity correlation function $g_{II}(s)$ for all simulations, plotted alongside $g_{II}(s)$ for binarized experimentally observed chromosome sections. In each plot, one faint curve corresponds to one run of a simulation, with boundary geometry and ellipse shape derived from a specific experimental data set, as shown in (a). Colored points with error bars represent averages of simulated data; black points with error bars show the average experimental $g_{II}(s)$ (Main Text Fig. 4(b)). All error bars indicate SE. (c) Histograms of $w_{\rm min}$ for all identified chromosomes in the binarized experimental images ($n = 216$ chromosomes in 11 spindles), the corresponding randomized chromosome sections, and the images generated by our repulsive ellipsoid simulations. (d) Table of chromosome configuration parameters for the binarized experimental images and all simulations. The randomized chromosome simulations lack local extrema in $g_{II}(s)$, so the first two parameters are not defined for this data set.

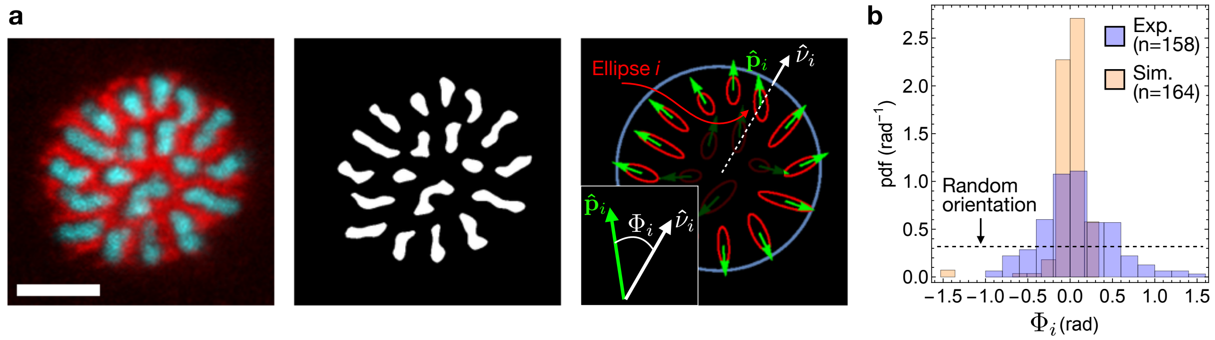

Figure S15: **The outermost ring of metaphase chromosomes is radially oriented.** (a) Left: Confocal micrograph of metaphase plate ($x = 0$) cross-section. Scale bar $5\,\mu$m. *Middle:* Binarization of chromsomes. *Right:* The orientation Φ_i of each chromosome is defined by the relation $\cos\Phi_i = \hat{\mathbf{p}}_i \cdot \hat{\nu}_i$, where $\hat{\mathbf{p}}_i$ is the long axis of best-fit ellipse i and $\hat{\nu}_i$ is the normal to the circle that best fits the spindle boundary, evaluated at the closest point to the center of ellipse i. Only chromosomes whose centers are more than $3.25\,\mu$m from the center of the spindle are included in subsequent analysis. (b) For both simulated and experimental data, the distribution of Φ_i is strongly peaked near zero, indicating that most chromosomes tend to align normal to the spindle boundary. The black dashed line indicates the expected distribution if the ellipses were randomly oriented. For experimental data, $n = 158$ boundary chromosomes from 11 spindles were analyzed; for simulated data, $n = 164$ boundary ellipses from 11 simulations were analyzed. Histogram shows only data from the r^{-3} simulation; other simulated data sets give similar results.

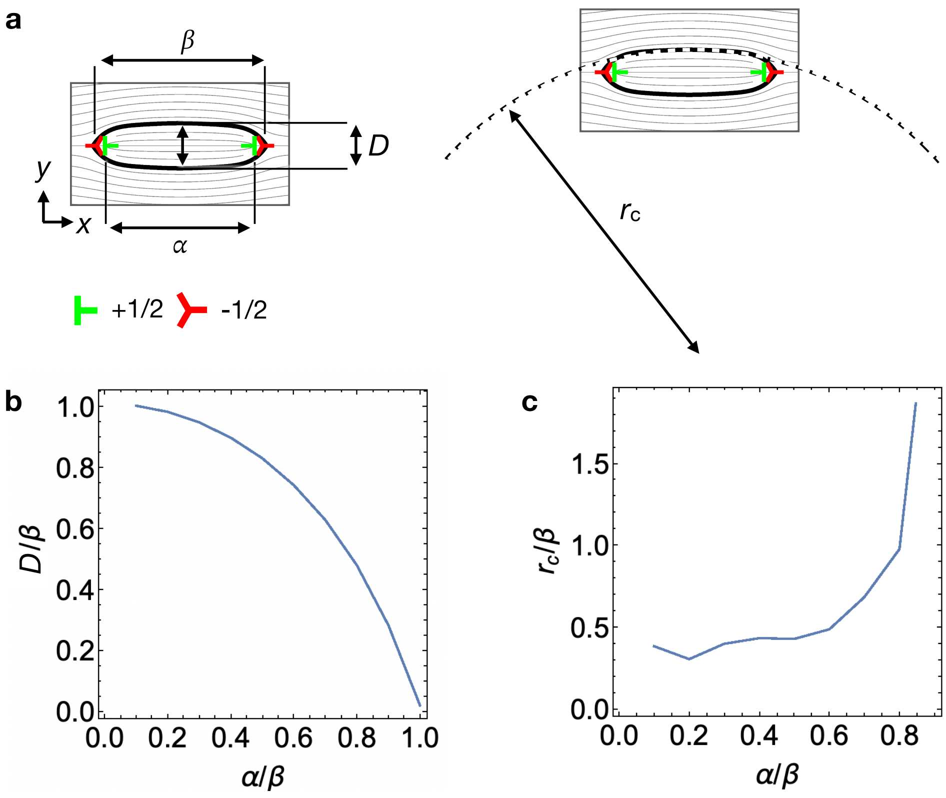

Figure S16: **Relationship between void boundary geometry and defect spacing.** (a) For a void in our 2D model, the defect spacing parameters α and β uniquely determine the void waist width D and radius of curvature r_c. (b & c) Plots of void aspect ratio D/β and waist curvature r_c/β as a function of inner defect spacing α.

REFERENCES AND NOTES

1. B. Alberts, A. Johnson, J. Lewis, D. Morgan, M. Raff, K. Roberts, P. Walter, *Molecular Biology of the Cell* (Garland Science, ed. 6, 2015).

2. M. I. Anjur-Dietrich, C. P. Kelleher, D. J. Needleman, Mechanical mechanisms of chromosome segregation. *Cells* **10**, 465 (2021).

3. U. S. Eggert, T. J. Mitchison, C. M. Field, Animal cytokinesis: From parts list to mechanisms. *Annu. Rev. Biochem.* **75**, 543–566 (2006).

4. B. Mogessie, K. Scheffler, M. Schuh, Assembly and positioning of the oocyte meiotic spindle. *Annu. Rev. Cell Dev. Biol.* **34**, 381–403 (2018).

5. H. Maiato, A. Gomes, F. Sousa, M. Barisic, Mechanisms of chromosome congression during mitosis. *Biology* **6**, 13 (2017).

6. E. Nazockdast, S. Redemann, Mechanics of the spindle apparatus. *Semin. Cell Dev. Biol.* **107**, 91–102 (2020).

7. D. Gerlich, J. Beaudouin, B. Kalbfuss, N. Daigle, R. Eils, J. Ellenberg, Global chromosome positions are transmitted through mitosis in mammalian cells. *Cell* **112**, 751–764 (2003).

8. S. J. Klaasen, M. A. Truong, R. H. van Jaarsveld, I. Koprivec, V. Štimac, S. G. de Vries, P. Risteski, Snježana Kodba, K. Vukušić, K. L. de Luca, J. F. Marques, E. M. Gerrits, B. Bakker, F. Foijer, J. Kind, I. M. Tolić, S. M. A. Lens, G. J. P. L. Kops, Nuclear chromosome locations dictate segregation error frequencies. *Nature* **607**, 604–609 (2022).

9. P. M. Chakin, T. C. Lubensky, *Principles of Condensed Matter Physics* (Cambridge Univ. Press, ed. 1, 2000).

10. R. S. Lillie, The physiology of cell-division.—I. Experiments on the conditions determining the distribution of chromatic matter in mitosis. *Am. J. Physiol. Legacy Content* **15**, 46–84 (1905).

11. J. D. Bernal, The physical basis of life. *Proc. Phys. Soc. B* **62**, 597–618 (1949).

12. N. G. Anderson, Cell division. ii. A theoretical approach to chromosomal movements and the division of the cell. *Q. Rev. Biol.* **31**, 243–269 (1956).

13. J. Brugués, D. Needleman, Physical basis of spindle self-organization. *Proc. Natl. Acad. Sci. U.S.A* **111**, 18496–18500 (2014).

14. T. L. Nguyen, S. Pradeep, R. L. Judson-Torres, J. Reed, M. A. Teitell, T. A. Zangle, Quantitative phase imaging: Recent advances and expanding potential in biomedicine. *ACS Nano* **16**, 11516–11544 (2022).

15. G. Flé, E. V. Houten, G. Rémillard-Labrosse, G. FitzHarris, G. Cloutier, Imaging the subcellular viscoelastic properties of mouse oocytes. *Proc. Natl. Acad. Sci. U.S.A.* **120**, e2213836120 (2023).

16. M. C. Marchetti, J. F. Joanny, S. Ramaswamy, T. B. Liverpool, J. Prost, M. Rao, R. A. Simha, Hydrodynamics of soft active matter. *Rev. Mod. Phys.* **85**, 1143–1189 (2013).

17. D. Oriola, F. Jülicher, J. Brugués, Active forces shape the metaphase spindle through a mechanical instability. *Proc. Natl. Acad. Sci. U.S.A.* **117**, 16154–16159 (2020).

18. L. Liu, R. Oldenbourg, J. R. Trimarchi, D. L. Keefe, A reliable, noninvasive technique for spindle imaging and enucleation of mammalian oocytes. *Nat. Biotechnol.* **18**, 223–225 (2000).

19. Z. Holubcová, M. Blayney, K. Elder, M. Schuh, Error-prone chromosome-mediated spindle assembly favors chromosome segregation defects in human oocytes. *Science* **348**, 1143–1147 (2015).

20. A. I. Mihajlović, G. FitzHarris, Segregating chromosomes in the mammalian oocyte. *Curr. Biol.* **28**, R895–R907 (2018).

21. W. Conway, R. Kiewisz, G. Fabig, C. P. Kelleher, H.-Y. Wu, M. Anjur-Dietrich, T. Mller-Reichert, D. J. Needleman, Self-organization of kinetochore-fibers in human mitotic spindles. *eLife* **11**, e75458 (2022).

22. H. Sato, G. W. Ellis, S. Inoué, Microtubular origin of mitotic spindle form birefringence. demon- stration of the applicability of wiener's equation. *J. Cell Biol.* **67**, 501–517 (1975).

23. R. Oldenbourg, E. D. Salmon, P. T. Tran, Birefringence of single and bundled microtubules. *Biophys. J.* **74**, 645–654 (1998).

24. R. Oldenbourg, Polarized light microscopy: Principles and practice. *Cold Spring Harb. Protoc.* **2013**, pdb.top078600 (2013).

25. A. DeBenedictis, T. J. Atherton, Shape minimisation problems in liquid crystals. *Liq. Cryst.* **43**, 2352–2362 (2016).

26. P.-X. Wang, M. J. MacLachlan, Liquid crystalline tactoids: Ordered structure, defective coalescence and evolution in confined geometries. *Philos Trans. A Math. Phys. Eng. Sci.* **376**, 20170042 (2018).

27. A. Kuhnhold, P. van der Schoot, Structure of nematic tactoids of hard rods. *J. Chem. Phys.* **156**, 104501 (2022).

28. P. D. Gennes, J. Prost, *Physics of Liquid Crystals* (Oxford Science Publications, ed. 2, 1993).

29. R. D. Williams, Two transitions in tangentially anchored nematic droplets. *J. Phys. A Math. Gen.* **19**, 3211–3222 (1986).

30. P. Prinsen, P. van der Schoot, Shape and director-field transformation of tactoids. *Phys. Rev. E* **68**, 021701 (2003).

31. Groupe d'Etude des Cristaux Liquides (Orsay), Dynamics of fluctuations in nematic liquid crystals. *J. Chem. Phys.* **51**, 816–822 (1969).

32. M. W. G. Schneider, B. A. Gibson, S. Otsuka, M. F. D. Spicer, M. Petrovic, C. Blaukopf, C. C. H. Langer, P. Batty, T. Nagaraju, L. K. Doolittle, M. K. Rosen, D. W. Gerlich, A mitotic chromatin phase transition prevents perforation by microtubules. *Nature* **609**, 183–190 (2022).

33. J. D. Bernal, I. Fankuchen, X-ray and crystallographic studies of plant virus preparations: I. Introduction and preparation of specimens ii. Modes of aggregation of the virus particles. *J. Gen. Physiol.* **25**, 111–146 (1941).

34. Y. A. Nastishin, H. Liu, T. Schneider, V. Nazarenko, R. Vasyuta, S. V. Shiyanovskii, O. D. Lavrentovich, Optical characterization of the nematic lyotropic chromonic liquid crystals: Light absorption, birefringence, and scalar order parameter. *Phys. Rev. E* **72**, 041711 (2005).

35. H. Almohammadi, S. Martinek, Y. Yuan, P. Fischer, R. Mezzenga, Disentangling kinetics from thermodynamics in heterogeneous colloidal systems. *Nat. Commun.* **14**, 607 (2023).

36. Y. Yi, N. A. Clark, Orientation of chromonic liquid crystals by topographic linear channels: Multi-stable alignment and tactoid structure. *Liq. Cryst.* **40**, 1736–1747 (2013).

37. S. Zhou, A. Sokolov, O. D. Lavrentovich, I. S. Aranson, Living liquid crystals. *Proc. Natl. Acad. Sci. U.S.A.* **111**, 1265–1270 (2014).

38. M. M. Genkin, A. Sokolov, I. S. Aranson, Spontaneous topological charging of tactoids in a living nematic. *New J. Phys.* **20**, 043027 (2018).

39. P. Poulin, H. Stark, T. C. Lubensky, D. A. Weitz, Novel colloidal interactions in anisotropic fluids. *Science* **275**, 1770–1773 (1997).

40. P. Sharma, A. Ward, T. Gibaud, M. F. Hagan, Z. Dogic, Hierarchical organization of chiral rafts in colloidal membranes. *Nature* **513**, 77–80 (2014).

41. C. Joslin, C. Gray, Multipole expansions in two dimensions. *Mol. Phys.* **50**, 329–345 (1983).

42. J. D. Jackson, *Classical Electrodynamics* (John Wiley & Sons, ed. 3, 2021).

43. V. Kesler, B. Murmann, H. T. Soh, Going beyond the debye length: Overcoming charge screening limitations in next-generation bioelectronic sensors. *ACS Nano* **14**, 16194–16201 (2020).

44. S. Cuylen, C. Blaukopf, A. Z. Politi, T. Mller-Reichert, B. Neumann, I. Poser, J. Ellenberg, A. A. Hyman, D. W. Gerlich, Ki-67 acts as a biological surfactant to disperse mitotic chromosomes. *Nature* **535**, 308–312 (2016).

45. R. Behringer, M. Gertsenstein, K. Vintersten, A. Nagy, *Manipulating the Mouse Embryo: A Laboratory Manual* (Cold Spring Harbor Laboratory Press, ed. 4, 2014).

46. X. Wu, Z. Han, X. Hao, Y. Zhao, C. Zhou, X. Wen, C. Liang, Combined use of dbcamp and ibmx minimizes the damage induced by a longterm artificial meiotic arrest in mouse germinal vesicle oocytes. *Mol. Reprod. Dev.* **87**, 262–273 (2020).

47. R. Gonzalez, R. Woods, *Digital Image Processing* (Pearson Education, ed. 4, 2018).

48. G. FitzHarris, J. Carroll, K. Swann, Electrical-assisted microinjection for analysis of fertilization and cell division in mammalian oocytes and early embryos. *Methods Cell Biol.* 431–440 (2018).

49. C. P. Kelleher, D. J. Needleman, Y. P. Rana, Data and codes for long-range repulsion between chromosomes in mammalian oocyte spindles. Zenodo (2024). https://doi.org/10.5281/zenodo.12806561.

50. C. P. Kelleher, D. J. Needleman, Y. P. Rana, Long-range repulsion between chromosomes in mammalian oocyte spindles. https://osf.io/p9t7z/.

51. M. T. Tran, R. Oldenbourg, Point spread function of the polarized light field microscope. *J. Opt. Soc. Am. A* **39**, 1095–1103 (2022).

52. H. H. Hopkins, P. M. Barham, The influence of the condenser on microscopic resolution. *Proc. Phys. Soc. B* **63**, 737–744 (1950).

53. O. Wiener, Theorie des mischkorpers fur das feld der stationaren stromung. *Abhandl. Sachs. Ges. Wiss. Math. Phys. K1*, 509–598 (1912).

54. F. M. Nixon, T. R. Honnor, N. I. Clarke, G. P. Starling, A. J. Beckett, A. M. Johansen, J. A. Brettschneider, I. A. Prior, S. J. Royle, Microtubule organization within mitotic spindles revealed by serial block face scanning em and image analysis. *J. Cell Sci.* **130**, 1845–1855 (2017).

55. J. B. Olmsted, G. G. Borisy, Microtubules. *Annu. Rev. Biochem.* **42**, 507–540 (1973).

56. B. Gul, S. Ashraf, S. Khan, H. Nisar, I. Ahmad, Cell refractive index: Models, insights, applications and future perspectives. *Photodiagnosis Photodyn. Ther.* **33**, 102096 (2021).

57. J. M. O. de Zarate, J. V. Sengers, *Hydrodynamic Fluctuations in Fluids and Fluid Mixtures* (Elsevier Science, ed. 1, 2006).

58. S. A. Edwards, J. M. Yeomans, Spontaneous flow states in active nematics: A unified picture. *Europhys. Lett.* **85**, 18008 (2009).

59. R. N. Bracewell, Numerical transforms. *Science* **248**, 697–704 (1990).

60. A. Satoh, "Typical properties of colloidal dispersions calculable by molecular-microsimulations" in *Studies in Interface Science* (Elsevier, 2003), pp. 153–159.

61. B. Li, D. Zhou, Y. Han, Assembly and phase transitions of colloidal crystals. *Nat. Rev. Mater.* **1**, 15011 (2016).

62. M. Leoni, O. V. Manyuhina, M. J. Bowick, M. C. Marchetti, Defect driven shapes in nematic droplets: Analogies with cell division. *Soft Matter* **13**, 1257–1266 (2017).

63. F. Vafa, M. J. Bowick, M. C. Marchetti, B. I. Shraiman, Multi-defect dynamics in active nematics. arXiv:2007.02947 [cond-mat.soft] (2020).
